# Diagnostic value of syndecan-1 for coronary artery lesions and correlation analysis of laboratory indicators in Kawasaki disease patients

**DOI:** 10.1186/s13052-024-01772-0

**Published:** 2024-10-09

**Authors:** Ling Dai, Lingbo Zhang, Jie He, Rui Huang, Wenwen Tang, Huan Guo, Xiaoke Shang

**Affiliations:** 1 ICU Department, Wuhan No.1 Hospital, Wuhan, Hubei China; 2 Cardiothoracic Surgery Department, The People’s Hospital of Lincang, No. 116 Nantang Road, Linxiang District, Lincang, Yunnan China; 3 Department of Oncology, Zhangqiu People’s Hospital, Jinan, Shandong China; 4 Department of Cardiology, Chen Zhou 3rd People’s Hospital, Chenzhou, Hunan China; 5 Department of Cardiology, Wuhan No. 9 Hospital, Wuhan, Hubei China; 6 Department of Cardiovascular Surgery, Union Hospital, Tongji Medical College, Huazhong University of Science and Technology, No. 1277 Jiefang Avenue, Jianghan District, Wuhan, Hubei Province China

**Keywords:** Syndecan-1, Coronary artery lesions, Kawasaki disease, Diagnostic, Correlation analysis

## Abstract

**Background:**

To explore the application value of syndecan-1 (SDC-1) in the diagnosis of coronary artery lesions (CALs) in Kawasaki disease (KD) patients and the correlation of multiple laboratory indicators in KD patients.

**Methods:**

86 pediatric Kawasaki disease (KD) patients and 52 healthy controls admitted from January 2018 to December 2023 were retrospectively analyzed. Venous blood samples from KD patients were analyzed for white blood cells (WBC), platelets (PLT), C-reactive protein (CRP), interleukin-6 (IL-6), syndecan-1 (SDC-1), coagulation parameters, and lipid profiles. Correlations between these laboratory indicators were assessed. Receiver operating characteristic (ROC) curve analysis determined the diagnostic value of SDC-1 for coronary artery lesions (CALs) in KD patients. SDC-1 levels were further compared across different CAL severity groups.

**Results:**

The levels of ALT, AST, WBC, PLT, CRP, IL-6, and SDC-1 in the KD group were significantly higher than those in the control group (*P* < 0.05). Coagulation function analysis showed that APTT, TT and FIB levels were significantly increased in the KD group compared with the control group (*P* < 0.05). Lipid profile analysis revealed that TC, HDL-C, and ApoA1 were significantly decreased, whereas TG, LDL-C, and ApoB100 were significantly increased in the KD group (*P* < 0.05). Refractory KD patients exhibited significantly higher levels of ALT, AST, SDC-1, CRP, WBC, and TG compared to responsive KD patients (*P* < 0.05). Correlation analysis indicated a strong positive correlation between PLT and LDL-C (*r* = 0.227, *P* = 0.035) and between IL-6 and TG (*r* = 0.491, *P* = 0.000), while CRP was negatively correlated with ApoA1 (*r* = -0.265, *P* = 0.014). Among the 86 KD patients, 41 (47.67%) developed CALs, with 19 classified as mild, 15 as moderate, and 7 as severe. For predicting CALs among KD patients, the threshold of SDC-1 was identified as 5.5 ng/ml, with a sensitivity of 70.7%, specificity of 64.4%, positive predictive value of 65.91%, negative predictive value of 69.05%, and an AUC of 0.762 (95% confidence interval 0.662–0.861, *P* < 0.001). SDC-1 levels significantly differed among the CAL severity groups (*P* = 0.008), with higher levels observed in moderate compared to mild CALs, and in severe compared to moderate CALs.

**Conclusion:**

In conclusion, SDC-1 has strong clinical value in the diagnosis of CALs in KD patients, and there is a close relationship between the levels of inflammatory factors, coagulation function and lipid levels in KD patients.

**Supplementary Information:**

The online version contains supplementary material available at 10.1186/s13052-024-01772-0.

## Introduction

Kawasaki disease (KD), also recognized as mucocutaneous lymph node syndrome, presents as an acute, self-limiting febrile illness without a clearly defined cause [1]. Its manifestation includes fever accompanied by distinctive mucocutaneous lesions, lymph node enlargement, and skin alterations [2]. This condition stands as a prominent vasculitic syndrome, primarily affecting the pediatric population and infants [3]. The coronary vascular system frequently falls under the impact of KD, resulting in distinctive changes such as thickening of the coronary arterial wall, luminal dilation, and in severe cases, the formation of coronary artery aneurysms, which is called coronary artery lesions (CALs) [4]. Notably, the incidence of heart disease caused by KD has surpassed rheumatic heart disease, emerging as a significant cause of acquired heart conditions among children [5].

Despite extensive research, the precise mechanisms underlying the development of CALs in KD remain elusive, mainly including inflammatory responses, oxidative stress, disturbances in lipid metabolism, abnormalities in coagulation and fibrinolysis systems, and impairment of endothelial function [6, 7]. At present, laboratory indicators used to assist the diagnosis of CALs mainly include lipid indexes, coagulation function indexes and inflammatory factors, but most of them lack specificity and are easy to be interfered by other factors. Syndecan-1 (SDC-1) is mainly expressed on the glycocalyx of epithelial cells, endothelial cells and plasma cells, and is an important transmembrane heparin sulfate proteoglycan in the Syndecan family [8], which is closely related to vascular endothelial damage. Previous studies have reported that SDC-1 levels are significantly elevated in KD patients with CALs [9, 10]. These studies have led to the hypothesis that patients with KD may exhibit abnormalities in various laboratory indicators, such as SDC-1, lipid levels, platelet count, C-reactive protein (CRP), and Interleukin-6 (IL-6), and whether these abnormalities are associated with the prognosis of KD patients. Exploring the correlation of these indicators helps to reveal their key role in assessing KD severity.

Herein, the purpose of this study was to explore the application value of syndecan-1 (SDC-1) in the diagnosis of CALs in KD patients and the correlation of multiple laboratory indicators in KD patients, so as to provide a theoretical basis for the diagnosis and management of CALs in KD patients in clinic.

## Materials and methods

### Clinical data

**Fig. 1:**
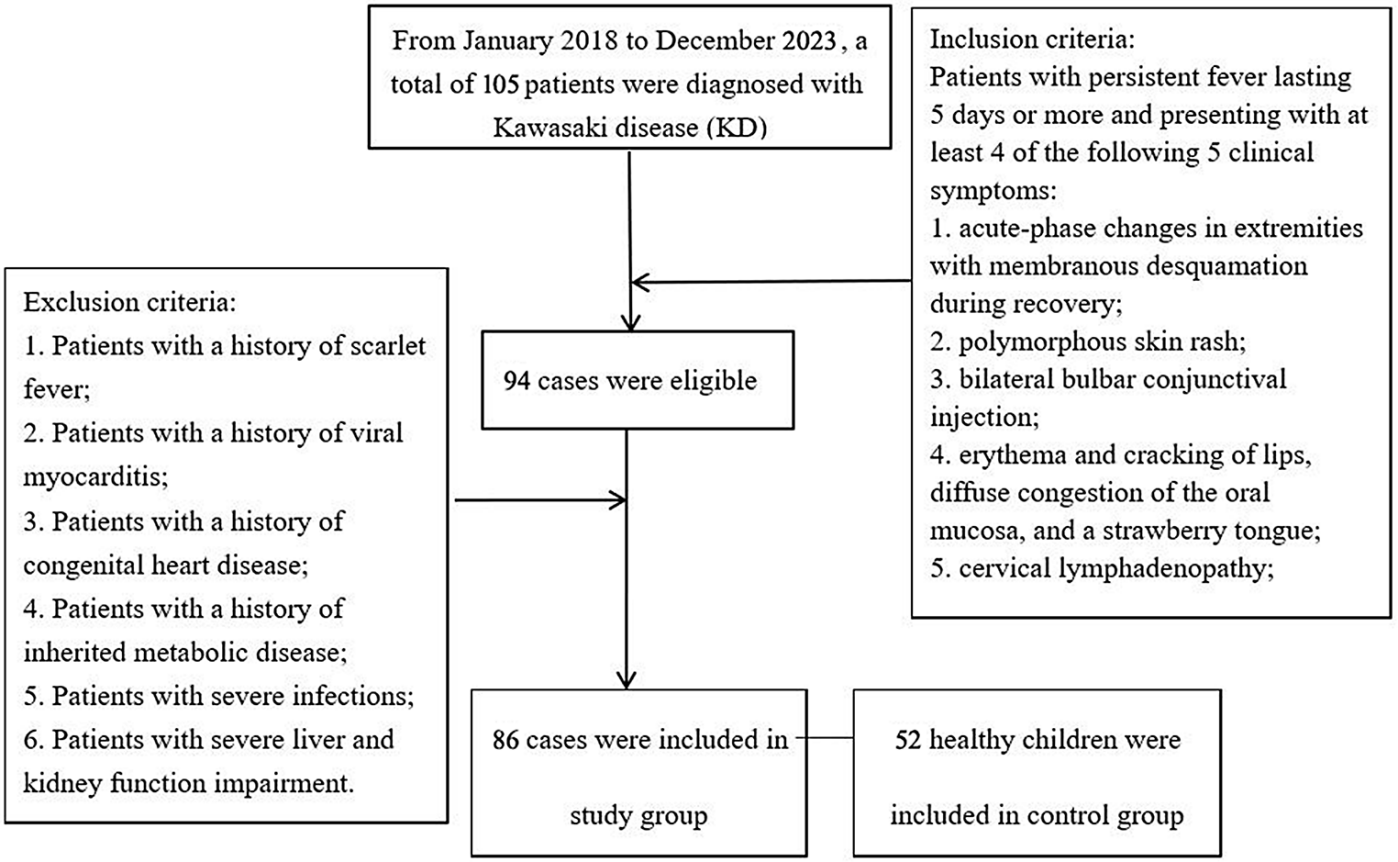
Flow chart of patient inclusion

From January 2018 to December 2023, 86 KD pediatric cases admitted to our hospital and other 52 healthy children were included in this retrospective study. All KD patients were classified as KD group (*n* = 86) and healthy children as control group (*n* = 52). This study protocol was formulated in accordance with the requirements of the Declaration of Helsinki of the World Medical Association. It was approved by our institution’s medical ethics committee (2017 Ethics Review (0216)) and the informed consent forms were obtained from all patients. The flow diagram for including patients was shown in Fig. 1.

### Inclusion criteria and exclusion criteria

Inclusion criteria: (1) The patient met the KD diagnostic criteria published by the American Heart Association (AHA) in 2017 [11]. Patients with persistent fever lasting 5 days or more and presenting with at least 4 of the following 5 clinical symptoms: ① acute-phase changes in extremities with membranous desquamation during recovery; ② polymorphous skin rash; ③ bilateral bulbar conjunctival injection; ④ erythema and cracking of lips, diffuse congestion of the oral mucosa, and a strawberry tongue; ⑤ cervical lymphadenopathy; (2) Patients who have signed informed consent;

Exclusion criteria: (1) Patients with a history of scarlet fever; (2) Patients with a history of viral myocarditis; (3) Patients with a history of congenital heart disease; (4) Patients with a history of inherited metabolic disease; (5) Patients with severe infections; (6) Patients with severe liver and kidney function impairment.

### Laboratory indicators and detection methods

Upon admission, venous blood samples were obtained from all KD patients for routine laboratory tests. The laboratory indicators included general laboratory indicators, coagulation function indicators and lipid indicators. General laboratory indicators included white blood cells (WBC), platelets (PLT), C-reactive protein (CRP), interleukin 6 (IL-6), SDC-1. The coagulation function indicators included thrombin time (TT), activated partial thromboplastin time (APTT), fibrinogen (FIB). The lipid indicators included total cholesterol (TC), triglycerides (TG), high-density lipoprotein cholesterol (HDL-C), low density lipoprotein cholesterol (LDL-C), apolipoprotein A1 (ApoA1) and apolipoprotein B100 (ApoB100).

Serum SDC-1 levels were assessed using the enzyme-linked immunosorbent assay (ELISA) method with the BioBase 2000 fully automatic enzyme immunoassay analyzer (Shandong Biotech Science Instrument Co., Ltd.). Coagulation indicators were measured using the Beckman Coulter ACL-200 fully automated coagulation analyzer, and the lipid profiles were analyzed using the Hitachi 7600-020 fully automated biochemical analyzer.

### Diagnostic criteria and classification for CALs

**Fig. 2:**
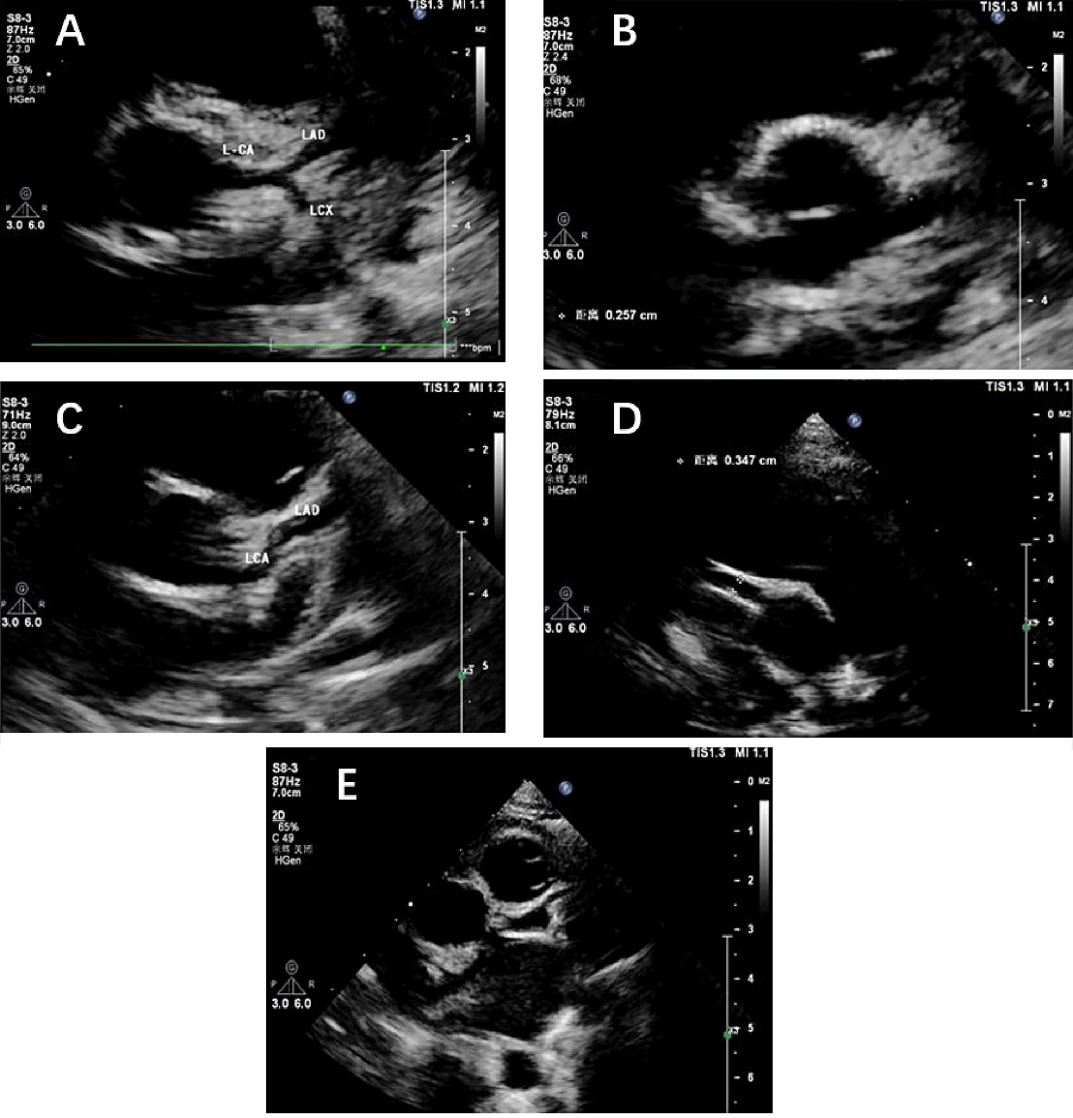
The figures of echocardiography. (**A**) Normal; (**B**) Dilatation of the left main coronary artery; (**C**) Dilatation of the left main coronary artery and the left anterior descending branch with thrombus formation; (**D**) Dilatation of the right coronary artery; (**E**) Localized aneurysm-like dilatation of the left anterior descending coronary artery

CALs in this study were evaluated using echocardiography. The imaging was performed with a [GE Vivid E95, manufactured by GE Healthcare], which was utilized to obtain measurements of coronary artery dimensions (Fig. 2). Z-scores were applied to coronary arteries with an internal diameter of ≥ 2.0 adjusted for body surface area. Whether in the right proximal coronary artery, the left coronary aorta, or the left anterior descending branch, CALs were diagnosed with a Z score calculated using the Dallaire equation. More than 1 month after the onset of the disease, patients with a maximum Z score of ≥ 2.5 were considered to have coronary aneurysm (CAA), and patients with a maximum Z score of 2.0-2.5 were considered to have coronary artery dilation [12, 13]. Patients with CALs were classified as mild CALs (small aneurysms under 5 mm), moderate CALs (large aneurysms between 5 and 8 mm), or severe CALs (giant aneurysms above 8 mm) based on the size of their dilated coronary artery diameter [14].

### Treatment protocol

All KD patients received intravenous immunoglobulin (IVIG) at a dose of 2 g/kg between days 4 and 10 of illness, following the American Heart Association (AHA) guidelines. This treatment was combined with medium- or high-dose aspirin (ASA). Patients presenting with severe coronary artery lesions (CALs) underwent initial corticosteroid intensification therapy, which involved the administration of intravenous methylprednisolone (IVMP) at a dose of 2 mg/kg/day, given twice daily, followed by a transition to oral prednisolone. For patients with refractory KD, a second IVIG treatment was administered.

### Statistical analysis

Data were analyzed using SPSS 22.0 software. The Kolmogorov-Smirnov test was employed for normality assessment. Non-normally distributed continuous variables were represented by median and quartiles, while normally distributed data were expressed as (x ± s). Independent sample t-tests or analysis of variance were used for intergroup comparisons of normally distributed data, and categorical data were presented as frequencies or rates (%), analyzed using the chi-square test. Receiver operating characteristic (ROC) curve was constructed and area under the curve (AUC) was calculated to evaluate the diagnostic value of SDC-1 laboratory indicators for CALs in KD patients. Statistical significance was set at *P* < 0.05.

## Results

### Baseline clinical characteristics

There were 86 subjects in the KD group, including 48 males (55.81%) and 38 females (44.19%). The age range was 3 months to 8.5 years, with a mean age of 3.34±1.59 years. There were 52 subjects in the control group, including 25 males (48.08%) and 27 females (51.92%). The age range was 4 months to 8.7 years, with a mean age of 3.48±1.60 years.

The levels of WBC, PLT, CRP, IL-6 and SDC-1 in KD group were significantly higher than those in control group (*P* < 0.05). In the index of coagulation function, APTT, TT and FIB levels were significantly increased in KD group compared with control group (*P* < 0.05). In terms of lipid indexes, compared with control group, TC, HDL-C and ApoA1 in KD group were significantly decreased, while TG, LDL-C and ApoB100 were significantly increased (*P* < 0.05). Additionally, in liver function parameters, AST and ALT levels in the KD group were significantly higher than those in the control group (*P* < 0.05). (Table 1). It is noteworthy that, compared with the refractory KD group, the levels of ALT, AST, SDC-1, CRP, WBC count and TG were significantly reduced in the responsive KD group (*P* < 0.05). (Table 2) There were no significant differences in other baseline clinical features (*P* > 0.05).

**Table 1:** Comparison of baseline clinical characteristics between KD group and control group

Clinical characteristics	KD group (*n* = 86)	Control group (*n* = 52)	χ^2^/t	*P*
Male (%)	48 (55.82%)	25 (48.08%)	0.779	0.378
Age (years)	3.34±1.59	3.48±1.60	-0.489	0.625
Weight (kg)	15.30±3.11	15.23±3.07	0.125	0.901
ALT(U/L)	77.14±23.46	26.35±10.37	14.748	**0.000** ^*^
AST(U/L)	65.71±17.80	28.81±8.73	13.957	**0.000** ^*^
SDC-1 (ng/mL)	5.52±1.02	2.51±0.65	19.030	**< 0.001** ^*^
CRP (mg/L)	62.53±23.88	5.69±1.60	18.012	**< 0.001** ^*^
IL-6 (pg/mL)	71.54±10.90	5.02±2.10	43.479	**< 0.001** ^*^
WBC (×10^9^/L)	15.76±2.28	6.57±0.88	27.830	**< 0.001** ^*^
PLT (×10^9^/L)	354.84±58.75	244.31±38.90	12.054	**< 0.001** ^*^
APTT (s)	33.02±5.75	21.23±1.64	14.430	**< 0.001** ^*^
TT (s)	16.14±2.99	14.40±2.65	3.442	**0.001** ^*^
FIB (g/L)	6.07±1.98	3.01±1.18	10.082	**0.000** ^*^
TC (mmol/L)	3.36±0.61	4.23±0.65	-7.849	**< 0.001** ^*^
TG (mmol/L)	1.62±0.54	0.87±0.25	9.419	**< 0.001** ^*^
HDL-C (mmol/L)	0.84±0.18	1.50±0.31	-15.789	**< 0.001** ^*^
LDL-C (mmol/L)	2.25±0.45	1.97±0.59	3.159	**0.002** ^*^
ApoA1 (g/L)	0.87±0.25	1.36±0.28	-10.614	**0.000** ^*^
ApoB100 (g/L)	0.98±0.33	0.77±0.18	4.104	**< 0.001** ^*^

*Note* * indicates a *P* value less than 0.05. Abbreviation: C-reactive protein (CRP), syndecan-1 (SDC-1), white blood cells (WBC), platelets (PLT), interleukin 6 (IL-6), thrombin time (TT), activated partial thromboplastin time (APTT), fibrinogen (FIB), total cholesterol (TC), triglycerides (TG), high-density lipoprotein cholesterol (HDL-C), low density lipoprotein cholesterol (LDL-C), apolipoprotein A1 (ApoA1) and apolipoprotein B100 (ApoB100)

**Table 2:** Comparison of baseline clinical characteristics between refractory KD group and responsive KD group

Clinical characteristics	Refractory KD (*n* = 31)	Responsive KD (*n* = 55)	χ^2^/t	*P*
Male (%)	20(51.61%)	28(43.64%)	1.488	0.223
Age (years)	3.36±1.65	3.33±1.57	0.105	0.917
Weight (kg)	15.44±3.17	15.22±3.11	0.871	0.756
ALT(U/L)	86.45±23.19	71.89±22.13	-2.879	**0.005** ^*^
AST(U/L)	72.71±16.65	61.76±17.34	-2.851	**0.005** ^*^
SDC-1 (ng/mL)	6.24±0.77	5.12±0.93	5.690	**0.000** ^*^
CRP (mg/L)	72.80±34.52	61.44±13.74	2.163	**0.033** ^*^
IL-6 (pg/mL)	74.12±13.53	70.09±8.90	1.665	0.100
WBC (×10^9^/L)	16.48±2.29	15.35±2.19	2.278	**0.025** ^*^
PLT (×10^9^/L)	363.48±56.85	349.96±59.75	1.025	0.308
APTT (s)	34.03±6.34	32.45±5.36	1.226	0.224
TT (s)	16.03±3.04	16.20±3.00	-0.248	0.805
FIB (g/L)	6.13±2.26	6.04±1.83	-0.214	0.831
TC (mmol/L)	3.31±0.66	3.39±0.59	-0.639	0.525
TG (mmol/L)	1.86±0.61	1.48±0.44	3.316	**0.001** ^*^
HDL-C (mmol/L)	0.83±0.19	0.84±0.18	-0.264	0.792
LDL-C (mmol/L)	2.27±0.42	2.23±0.47	0.377	0.707
ApoA1 (g/L)	0.87±0.25	0.88±0.25	-0.185	0.853
ApoB100 (g/L)	1.05±0.39	0.93±0.29	1.643	0.104

*Note* * indicates a *P* value less than 0.05. Abbreviation: C-reactive protein (CRP), syndecan-1 (SDC-1), white blood cells (WBC), platelets (PLT), interleukin 6 (IL-6), thrombin time (TT), activated partial thromboplastin time (APTT), fibrinogen (FIB), total cholesterol (TC), triglycerides (TG), high-density lipoprotein cholesterol (HDL-C), low density lipoprotein cholesterol (LDL-C), apolipoprotein A1 (ApoA1) and apolipoprotein B100 (ApoB100)

### Correlation analysis of general laboratory indicators and lipid indicators

In the correlation analysis, we primarily examined the relationships between general laboratory indicators and lipid profiles. The results demonstrated a strong positive correlation between PLT and LDL-C (*r* = 0.227, *P* = 0.035) as well as IL-6 and TG (*r* = 0.491, *P* = 0.000). A moderate positive correlation was observed between SDC-1 and TG (*r* = 0.250, *P* = 0.020) and between IL-6 and ApoB100 (*r* = 0.253, *P* = 0.019). Additionally, there was a moderate negative correlation between CRP and ApoA1 (*r* = -0.265, *P* = 0.014), with a stronger positive correlation observed between CRP and ApoB100 (*r* = 0.404, *P* = 0.000). Moreover, a stronger correlation was found between IL-6 and TG (*r* = 0.491, *P* = 0.000) (Table 3).

**Table 3:** Correlation analysis of general laboratory indicators and lipid indicators

	CRP (mg/L)		SDC-1		PLT (×10^9^/L)		IL-6 (pg/mL)	
	*r*	*P*	*r*	*P*	*r*	*P*	*r*	*P*
WBC(×10^9^/L)	0.135	0.217	0.035	0.749	0.085	0.438	-0.032	0.773
FIB(g/L)	**0.666**	**0.000** ^*^	-0.095	0.385	0.056	0.605	**-0.072**	**0.508**
APTT (s)	0.150	0.168	-0.039	0.722	0.018	0.873	**0.216**	**0.045** ^*^
TT (s)	-0.097	0.372	0.035	0.751	0.080	0.466	0.009	0.932
TC (mmol/L)	0.001	0.990	-0.047	0.664	-0.008	0.944	-0.155	0.155
TG (mmol/L)	**0.223**	**0.039** ^*^	**0.250**	**0.020** ^*^	0.002	0.987	**0.491**	**0.000** ^*^
HDL-C (mmol/L)	0.048	0.659	-0.149	0.171	0.025	0.817	0.022	0.839
LDL-C (mmol/L)	**-0.243**	**0.024** ^*^	0.000	0.993	**0.227**	**0.035**	0.004	0.972
ApoA1 (g/L)	**-0.265**	**0.014** ^*^	-0.106	0.330	-0.047	0.670	0.171	0.115
ApoB100 (g/L)	**0.404**	**0.000** ^*^	**0.223**	**0.039** ^*^	-0.097	0.374	**0.253**	**0.019** ^*^

*Note* * indicates a P value less than 0.05. Abbreviation: C-reactive protein (CRP), syndecan-1 (SDC-1), platelets (PLT), interleukin 6 (IL-6), white blood cells (WBC), fibrinogen (FIB), activated partial thromboplastin time (APTT), thrombin time (TT), total cholesterol (TC), triglycerides (TG), high-density lipoprotein cholesterol (HDL-C), low density lipoprotein cholesterol (LDL-C), apolipoprotein A1 (ApoA1) and apolipoprotein B100 (ApoB100)

### Application value of SDC-1 in diagnosis of CALs among KD patients

**Fig. 3:**
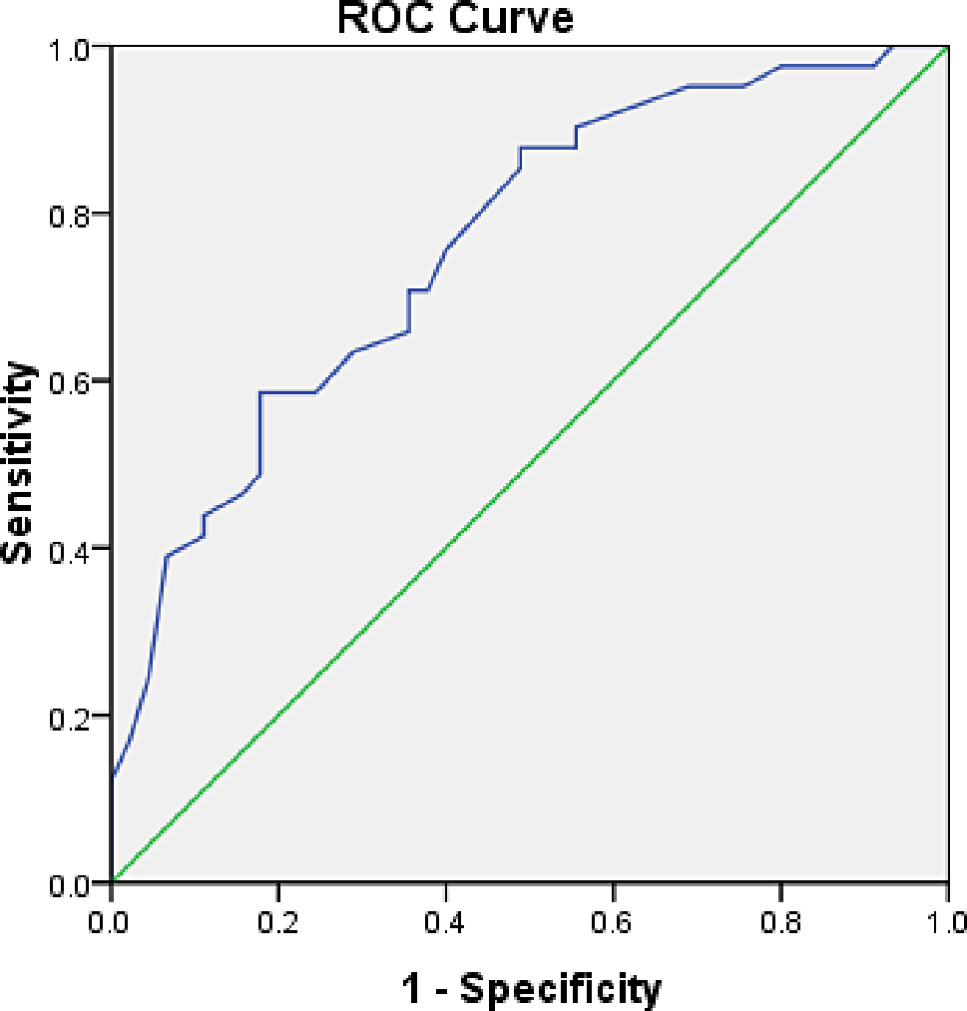
ROC curve of SDC-1 diagnosis in CALs patients among KD patients. Abbreviation: Receiver operating characteristic (ROC), syndecan-1 (SDC-1), coronary artery lesions (CALs), Kawasaki disease (KD)

Among 86 KD patients in this study, 41 (47.67%) developed CALs. For predicting CALs among KD patients, the threshold of SDC-1 was identified as 5.5 ng/ml, with a sensitivity of 70.7%, specificity of 64.4%, positive predictive value of 65.91%, negative predictive value of 69.05%, and an AUC of 0.762 (95% confidence interval 0.662–0.861, *P* < 0.001). The ROC curve of SDC-1 diagnosis in CALs patients among KD patients was presented in Fig. 3.

### SDC-1 levels in patients with different grades of CALs

Of the 41 patients with CALs, 19 (46.34%) had mild CALs, 15 (36.59%) had moderate CALs, and 7 (17.07%) had severe CALs. Their mean coronary artery diameters were 4.19 ± 2.80 mm, 6.57 ± 3.18 mm, and 9.30 ± 2.55 mm, respectively. SDC-1 levels were significantly different among the three groups (*P* < 0.05), among which the moderate CALs group was significantly higher than the mild CALs group (*P* < 0.05), and the severe CALs group was significantly higher than the moderate CALs group (*P* < 0.05). (Table 4)

**Table 4:** SDC-1 levels in patients with different grades of CALs

	Mild CALs group (*n* = 19)	Moderate CALs group (*n* = 15)	Severe CALs group (*n* = 7)	F	*P*
Coronary artery diameter (mm)	4.19 ± 2.80^*^	6.57 ± 3.18^*^^#^	9.30 ± 2.55^#^	8.468	0.001
SDC-1 (ng/mL)	5.52±0.87^*^	6.18±0.54^*#^	6.91±0.19^#^	5.464	0.008

*Note* * and # indicate a significant difference in the comparison. Abbreviation: syndecan-1 (SDC-1), coronary artery lesions (CALs)

## Discussion

KD stands as a prominent cause of acquired cardiac complications among children, often resulting in diverse CALs encompassing coronary artery dilation, aneurysm formation, fistulas, arterial reconstruction, and, in rare cases, thrombosis, stenosis, or occlusion [15]. These complications pose severe threats such as myocardial ischemia, infarction, and sudden death, gravely compromising the safety of affected children [16].

Disorders in lipid metabolism and coagulation function are prevalent in KD [17]. KD patients commonly exhibit altered lipid profiles, typified by reduced TC and HDL-C, alongside elevated levels of TG and LDL-C [18], which is consistent with the results of this study. The previous research suggested a potential correlation between lipid metabolism derangements in KD patients and inflammatory responses [19]. The heightened production of inflammatory cytokines in KD patients impedes the activity of lipoprotein lipase and hepatic lipase, stimulating the liver to secrete increased quantities of very low-density lipoprotein (VLDL), predominantly composed of TG [20]. Moderately elevated TG levels are often indicative of an increase in VLDL and its remnants. Moreover, reduced levels of HDL-C, primarily synthesized in the liver, contribute to elevated TG levels in KD patients, hindering the efficient transport of TG from extrahepatic tissues to the liver for metabolism [21]. ApoB100 serves as the primary apolipoprotein of LDL, while ApoA1 functions as the primary apolipoprotein of HDL [21]. Studies suggested an increased risk of coronary heart disease with elevated ApoB100 levels or reduced ApoA1 concentrations [21]. Our study identified significantly elevated levels of AST and ALT in patients with refractory KD compared to the responsive KD. The liver’s central role in immune modulation and systemic metabolism makes it particularly susceptible to inflammatory insults, which may exacerbate the overall disease severity in Kawasaki disease. From a mechanistic standpoint, the observed hepatic dysfunction in the refractory KD group likely reflects a heightened inflammatory burden. Kawasaki disease pathogenesis involves systemic vasculitis and endothelial activation, processes that can significantly impact hepatic function due to the liver’s role in detoxification and cytokine metabolism. The elevated hepatic enzymes observed may be indicative of an exacerbated inflammatory response, with the liver potentially acting as both a target and a mediator of systemic inflammation. This is further supported by the increased levels of pro-inflammatory cytokines, such as CRP and IL-6, which have been implicated in both liver injury and the pathophysiology of Kawasaki disease.

In addition to lipid metabolism disturbances, KD often manifests disruptions in coagulation function, reflected in abnormal coagulation parameters and platelet levels [22, 23]. In this study, compared with the control group, patients in the KD group showed significantly higher APTT, TT, PLT and FIB. The abnormal coagulation parameters and PLT levels observed in KD patients are often considered to be associated with heightened immune system activity [24]. The release of inflammatory factors within KD patients, such as CRP, IL-6, and tumor necrosis factor-alpha (TNF-a), induces vascular endothelial cell damage, exposing vascular wall collagen [25]. This exposure promotes platelet adhesion and activation, escalating platelet levels and exacerbating the coagulation state, intensifying vascular injury in KD patients [25]. Organs, particularly those rich in vascular supply, are susceptible to vasculitis effects in KD patients [26]. Studies highlighted the common occurrence of hepatic vasculitis and biliary system damage in KD patients, leading to impaired liver function [26]. Given the liver’s critical role in regulating coagulation function, impaired liver function results in decreased synthesis of coagulation factors and heparinase, along with reduced clearance of tissue thromboplastin and activated fibrinolysis factors [27]. This impairment may hinder vitamin K absorption, ultimately causing coagulation function abnormalities [25]. On the other hand, other references showed that systemic inflammation couldn’t fully explain hepatic impairments [28]. Endothelial cell damage and coagulation dysfunction contribute to CALs, significantly impacting the prognosis of KD patients [10].

The current study results showed that there was a strong positive correlation between IL-6 and TG in KD patients, suggesting that inflammatory cytokines are closely related to abnormal lipid metabolism. In addition, PLT also had a strong positive correlation with LDL-C. These complex associations suggested potential pathophysiological links between inflammatory factors, coagulation, and lipids in KD patients. The interplay between dysregulated lipid metabolism and coagulation function likely exacerbated the occurrence of CALs.

Syndecan-1 (SDC-1) is a transmembrane heparan sulfate proteoglycan predominantly found on the glycocalyx of epithelial and endothelial cells. Its ectodomain can be cleaved by matrix metalloproteinases and released into the serum, where elevated levels serve as biomarkers for endothelial activation and damage in various conditions.

SDC-1 is involved in various cellular processes, including cell proliferation, apoptosis, and inflammation. It plays a crucial role in modulating the vascular endothelial barrier function and inflammatory response, both of which are central to the pathophysiology of KD. SDC-1 interacts with several signaling molecules, including growth factors, cytokines, and chemokines, facilitating their binding to their respective receptors, thereby modulating downstream signaling pathways such as the MAPK/ERK [29] and NF-κB pathways [30]. These pathways are known to influence inflammatory processes and endothelial cell function, which are critical in the development of CALs.

In KD, the endothelial damage and increased vascular permeability contribute to the formation of coronary aneurysms. SDC-1 shedding, induced by pro-inflammatory cytokines, results in the release of its ectodomain, which can act as a soluble receptor, further exacerbating the inflammatory response and endothelial dysfunction. The loss of SDC-1 from the endothelial surface also leads to a breakdown of the glycocalyx, a protective layer on the endothelial cells, making the coronary arteries more susceptible to damage and aneurysm formation.

In our study, we have observed that elevated levels of SDC-1 are correlated with the severity of CALs in KD patients, suggesting that SDC-1 may serve as a potential biomarker for predicting the development and progression of CALs. The detailed mechanistic insights into SDC-1’s role in KD provided in the revised manuscript aim to offer a more comprehensive understanding of its involvement in disease pathogenesis and its potential as a therapeutic target.

SDC-1 plays a key role in various inflammatory disease processes, and an elevated level usually means an increased inflammatory response [26]. During inflammatory states, the extracellular domain of SDC-1 on endothelial cell surfaces is cleaved by various mediators, such as cytokines or reactive oxygen species (ROS), releasing it into the serum. Hence, increased serum SDC-1 levels indicate endothelial dysfunction [31]. Studies comparing KD patients with healthy children have found significantly elevated serum SDC-1 levels in KD patients, particularly those with CALs, suggesting SDC-1 as a potential biomarker for predicting CALs in KD [9]. Additionally, comparative studies have revealed markedly higher acute-phase serum SDC-1 levels in KD patients with CALs, further indicating SDC-1 as a predictive biomarker for CALs in KD patients [10]. ROC curve analysis in this study showed that SDC-1 was a reliable predictor of CALs in KD patients with high sensitivity and specificity and was a promising predictive marker.

ROC curve analysis revealed SDC-1 as a robust predictive factor for CALs in KD patients, demonstrating high sensitivity and specificity. Hence, SDC-1 emerges as a promising novel biomarker in KD patients, aiding in diagnosing coronary artery damage and offering invaluable insights for clinicians. In addition, we also found that SDC-1 levels were significantly different in patients with different severity of CALs, suggesting that SDC-1 consideration could be combined with other auxiliary inspections to determine the stage of KD. In our study, we conducted statistical analyses on factors such as age, gender and weight, and found no statistically significant differences among the groups in our study. This suggests that the impact of these variables on the study outcomes within our sample may be limited.

Limitations of this study include its retrospective design and relatively small sample size. As with any retrospective analysis, there may be inherent biases and limitations in data collection and analysis. The sample size, though sufficient for initial exploration, may limit the generalizability of the findings to broader populations of KD patients. Larger prospective studies are warranted to validate the findings and further elucidate the diagnostic utility of SDC-1 and its correlation with CALs in KD. On the other hand, the diversity and variability of treatment status present challenges in controlling for this variable in the current analysis. What’s more, due to constraints on funding and resources, our study did not collect detailed data on disease duration. In future research, we will consider designing prospective studies to gather more comprehensive data, including regular updates on disease duration. We also plan to include treatment response as a covariate in the analysis to assess its potential impact on the outcomes. Additionally, while this study identified significant associations between SDC-1 levels and CAL severity, the underlying mechanisms driving these associations remain to be fully elucidated. Further research is needed to clarify the biological pathways involved and to assess the potential impact of confounding variables not accounted for in this study.

## Conclusion

In conclusion, SDC-1 has strong clinical value in the diagnosis of CALs in KD patients, and there is a close relationship between the levels of inflammatory factors, coagulation function and lipid levels in KD patients.

## Electronic supplementary material

Below is the link to the electronic supplementary material.

Supplementary Material 1

## Data Availability

The data and materials used and/or analysed during the current study are available from the corresponding author on reasonable request.

## Abbreviations

AHA: American Heart Association
ApoA1: Apolipoprotein A1
ApoB100: Apolipoprotein B100
APTT: Activated Partial Thromboplastin Time
AUC: Area Under The Curve
CAA: Coronary Aneurysm
CALs: Coronary Artery Lesions
CRP: C-Reactive Protein
ELISA: Enzyme-Linked Immunosorbent Assay
FIB: Fibrinogen
HDL-C: High-Density Lipoprotein Cholesterol
IL-6: Interleukin 6
KD: Kawasaki Disease
LDL-C: Low Density Lipoprotein Cholesterol
PLT: Platelets
ROC: Receiver Operating Characteristic
ROS: Reactive Oxygen Species
SDC-1: Syndecan-1
TC: Total Cholesterol
TG: Triglycerides
TNF-a: Tumor Necrosis Factor-Alpha
TT: Thrombin Time
VLDL: Very Low-Density Lipoprotein
WBC: White Blood Cells
