# Supplementary material for: Diagnostic value of syndecan-1 for coronary artery lesions and correlation analysis of laboratory indicators in Kawasaki disease patients
Source: Ital J Pediatr. 2024 Oct 9;50:209. doi: 10.1186/s13052-024-01772-0 (PMC11463127; doi:10.1186/s13052-024-01772-0)
Supplement: Supplementary file 1 — Supplementary Material 1 [file 13052_2024_1772_MOESM1_ESM.docx]

Dear Dr. Bozzola,

Thank you for the opportunity to revise our manuscript entitled "Diagnostic value of syndecan-1 for coronary artery lesions and correlation analysis of laboratory indicators in Kawasaki disease patients" (ITJP-D-24-00346). We appreciate the insightful comments and suggestions provided by the reviewers, which have greatly enhanced the clarity and quality of our work.

We have carefully addressed all the points raised by the reviewers and have made the necessary revisions to our manuscript. Below is a point-by-point response to each comment:

Reviewer reports:

Reviewer #2: The manuscript is well written, organized and comprehensive, All authors did a good work in this study. The Idea is - to some extent- new and would add to the literature
Some points should be addressed

¶Introduction section:

1.  Regarding this statements "In addition, we found that KD patients often have abnormalities in a variety of laboratory indicators, including SDC1, lipid levels, platelet counts, C-reactive protein, and IL-6." I think in the section of introduction, authors shouldn't mention or address their findings.

Reply: Thank you for the valuable suggestion, which is highly appreciated. We strongly agree with your opinion, the relevant sentence has been deleted in the revised manuscript as suggested.

2.  Instead of previous statements, authors should mention Hypothesis of this study in clear manner based on literature of review and recent studies

Reply: Thank you for the valuable suggestion, which help imporove the accuracy of the manuscript. We have rewrote the sentence instead of previous statements as suggeted.

**Changs in the text:**

These studies have led to the hypothesis that patients with KD may exhibit abnormalities in various laboratory indicators, such as SDC-1, lipid levels, platelet count, C-reactive protein (CRP), and Interleukin-6 (IL-6), and whether these abnormalities are associated with the prognosis of KD patients. (page 3-4, line 90-94)

¶Literature of Review

1.  The article provides a reasonably comprehensive overview of the existing literature on the cardiac complications and associated metabolic disturbances in Kawasaki disease.
2.  SDC-1 pathways and mechanisms should be more detailed discussed.
Reply: Thank you for your valuable feedback. We have revised the manuscript to include a more detailed discussion of the syndecan-1 (SDC-1) pathways and mechanisms in Kawasaki disease (KD), particularly focusing on their role in the development of coronary artery lesions (CALs) as suggested.

**Changs in the text:**

Syndecan-1 (SDC-1) is a transmembrane heparan sulfate proteoglycan predominantly found on the glycocalyx of epithelial and endothelial cells. Its ectodomain can be cleaved by matrix metalloproteinases and released into the serum, where elevated levels serve as biomarkers for endothelial activation and damage in various conditions.

SDC-1 is involved in various cellular processes, including cell proliferation, apoptosis, and inflammation. It plays a crucial role in modulating the vascular endothelial barrier function and inflammatory response, both of which are central to the pathophysiology of KD. SDC-1 interacts with several signaling molecules, including growth factors, cytokines, and chemokines, facilitating their binding to their respective receptors, thereby modulating downstream signaling pathways such as the MAPK/ERK^29^ and NF-κB pathways ^30^. These pathways are known to influence inflammatory processes and endothelial cell function, which are critical in the development of CALs.

In KD, the endothelial damage and increased vascular permeability contribute to the formation of coronary aneurysms. SDC-1 shedding, induced by pro-inflammatory cytokines, results in the release of its ectodomain, which can act as a soluble receptor, further exacerbating the inflammatory response and endothelial dysfunction. The loss of SDC-1 from the endothelial surface also leads to a breakdown of the glycocalyx, a protective layer on the endothelial cells, making the coronary arteries more susceptible to damage and aneurysm formation.

In our study, we have observed that elevated levels of SDC-1 are correlated with the severity of CALs in KD patients, suggesting that SDC-1 may serve as a potential biomarker for predicting the development and progression of CALs. The detailed mechanistic insights into SDC-1's role in KD provided in the revised manuscript aim to offer a more comprehensive understanding of its involvement in disease pathogenesis and its potential as a therapeutic target. (page 10-11, line 285-310)

**References**

29. Sun H, Hu Y, Gu Z, Owens RT, Chen YQ, Edwards IJ. Omega-3 fatty acids induce apoptosis in human breast cancer cells and mouse mammary tissue through syndecan-1 inhibition of the MEK-Erk pathway. Carcinogenesis. 2011 Oct;32(10):1518-24.

30. Lin L, Qiu D, Yang F, Xia Y, Cai S, Liao X, Deng W, Wu C. Hydrogen-rich saline upregulates the Sirt1/NF-κB signaling pathway and reduces vascular endothelial glycocalyx shedding in sepsis-induced acute kidney injury. Shock. 2024 May 27. (page 17, line 490-497)

¶Methodology

1.  Comprehensive and illustrated in a good manner.

Reply: Thank you for the positive comment, which is highly appreciated. We will continue our efforts in the follow-up study and look forward to your professional comments again.

2.  Patients with autoimmune diseases should be excluded (SDC-1 is affected in those patients)

Reply: Thank you for the professional suggestion, which help improve the accuracy of the manuscript. We have added the relevant content into the exclusion criteria.

**Changs in the text:**

7.Patients with autoimmune diseases. (page 5, line 123-124)

3.  Why not mention the stage of KD (either acute, subacute, or chronic). I think that measuring SDC-1 in different stages and classifying patients into subgroups based on stages would add more strength to the study.

Reply: Thank you for the valuable suggestion, which is highly appreciated. In the study, patients with Kawasaki Disease (KD, n=86) were categorized based on the severity of coronary artery involvement into no coronary artery injury group (n=44), mild coronary artery lesions (mild CALs, n=18), moderate coronary artery lesions (moderate CALs, n=17), and severe coronary artery lesions (severe CALs, n=7). Of course, we could also retrospectively classify KD into acute, subacute, or chronic phases according to the reviewers' suggestions. However, after thorough discussion and research, the research team believes that this may not be as closely aligned with the central theme of the article as categorizing into subgroups based on coronary artery injury. Therefore, we later included additional cases and also added subgroups for refractory KD and non-refractory KD

**Changs in the text:**

**Methods**: 86 pediatric Kawasaki disease (KD) patients and 52 healthy controls admitted from January 2018 to December 2023 were retrospectively analyzed. Venous blood samples from KD patients were analyzed for white blood cells (WBC), platelets (PLT), C-reactive protein (CRP), interleukin-6 (IL-6), syndecan-1 (SDC-1), coagulation parameters, and lipid profiles. Correlations between these laboratory indicators were assessed. Receiver operating characteristic (ROC) curve analysis determined the diagnostic value of SDC-1 for coronary artery lesions (CALs) in KD patients. SDC-1 levels were further compared across different CAL severity groups.

**Results**: The levels of ALT, AST, WBC, PLT, CRP, IL-6, and SDC-1 in the KD group were significantly higher than those in the control group (P<0.05). Coagulation function analysis showed that APTT and TT were significantly increased, while FIB levels were significantly decreased in the KD group compared with the control group (P<0.05). Lipid profile analysis revealed that TC, HDL-C, and ApoA1 were significantly decreased, whereas TG, LDL-C, and ApoB100 were significantly increased in the KD group (P<0.05). Refractory KD patients exhibited significantly higher levels of ALT, AST, CRP, WBC, and TG compared to responsive KD patients (P<0.05). Correlation analysis indicated a strong positive correlation between PLT and LDL-C (r=0.813, P<0.001) and between IL-6 and TG (r=0.926, P<0.001), while CRP was negatively correlated with ApoA1 (r=-0.599, P=0.000). Among the 86 KD patients, 41 (47.67%) developed CALs, with 19 classified as mild, 15 as moderate, and 7 as severe. For predicting CALs among KD patients, the threshold of SDC-1 was identified as 5.5 ng/ml, with a sensitivity of 70.7%, specificity of 64.4%, positive predictive value of 65.91%, negative predictive value of 69.05%, and an AUC of 0.762 (95% confidence interval 0.662-0.861, P<0.001). SDC-1 levels significantly differed among the CAL severity groups (P<0.05), with higher levels observed in moderate compared to mild CALs, and in severe compared to moderate CALs (P<0.05). (page 2-3, line 35-61)

There were 86 subjects in the KD group, including 48 males (55.81%) and 38 females (44.19%). The age range was 3 months to 8.5 years, with a mean age of 3.34±1.59 years. There were 52 subjects in the control group, including 25 males (48.08%) and 27 females (51.92%). The age range was 4 months to 8.7 years, with a mean age of 3.48±1.60 years. (page 6-7, line 180-184)

Additionally, in liver function parameters, AST and ALT levels in the KD group were significantly higher than those in the control group (P<0.05). (Table 1). It is noteworthy that, compared with the refractory KD group, the levels of ALT, AST, CRP, WBC count and TG were significantly reduced in the responsive KD group (P<0.05). (Table 2) (page 7, line 191-195)

In the correlation analysis, we primarily examined the relationships between general laboratory indicators and lipid profiles. The results demonstrated a strong positive correlation between PLT and LDL-C (r=0.813, P<0.001) as well as IL-6 and TG (r=0.926, P<0.001). A moderate positive correlation was observed between SDC-1 and TG (r=0.485, P=0.034) and between IL-6 and ApoB100 (r=0.487, P=0.033). Additionally, there was a moderate negative correlation between CRP and ApoA1 (r=-0.487, P=0.033), with a stronger negative correlation observed between CRP and ApoA1 (r=-0.599, P=0.000). Moreover, a weaker correlation was found between IL-6 and TG (r=0.229, P=0.034) (Table 3) (page 7, line 198-206)

Among 86 KD patients in this study, 41 (47.67%) developed CALs. For predicting CALs among KD patients, the threshold of SDC-1 was identified as 5.5 ng/ml, with a sensitivity of 70.7%, specificity of 64.4%, positive predictive value of 65.91%, negative predictive value of 69.05%, and an AUC of 0.762 (95% confidence interval 0.662-0.861, P<0.001). The ROC curve of SDC-1 diagnosis in CALs patients among KD patients was presented in Figure 3. (page 7-8, line 208-213)

Of the 41 patients with CALs, 19 (46.34%) had mild CALs, 15 (36.59%) had moderate CALs, and 7 (17.07%) had severe CALs. Their mean coronary artery diameters were 4.19±2.80 mm, 6.57±3.18 mm, and 9.30±2.55 mm (page 8, line 215-217)

Table 1. Comparison of baseline clinical characteristics between KD group and control group

| Clinical characteristics | KD group (n=86) | Control group (n=52) | χ2/t | P |
| --- | --- | --- | --- | --- |
| Male (%) | 48（55.82%） | 25（48.08%） | 0.779 | 0.378 |
| Age (years) | 3.34±1.59 | 3.48±1.60 | -0.489 | 0.625 |
| Weight (kg) | 15.30±3.11 | 15.23±3.07 | 0.125 | 0.901 |
| ALT(U/L) | 77.14±23.46 | 26.35±10.37 | 14.748 | 0.000 |
| AST(U/L) | 65.71±17.80 | 28.81±8.73 | 13.957 | 0.000 |
| SDC-1 (ng/mL) | 5.52±1.02 | 2.51±0.65 | 19.030 | <0.001* |
| CRP (mg/L) | 62.53±23.88 | 5.69±1.60 | 18.012 | <0.001* |
| IL-6 (pg/mL) | 71.54±10.90 | 5.02±2.10 | 43.479 | <0.001* |
| WBC (×109/L) | 15.76±2.28 | 6.57±0.88 | 27.830 | <0.001* |
| PLT (×109/L) | 354.84±58.75 | 244.31±38.90 | 12.054 | <0.001* |
| APTT (s) | 33.02±5.75 | 21.23±1.64 | 14.430 | <0.001* |
| TT (s) | 16.14±2.99 | 14.40±2.65 | 3.442 | 0.001 |
| FIB (g/L) | 2.53±1.01 | 4.91±1.43 | -11.455 | <0.001* |
| TC (mmol/L) | 3.36±0.61 | 4.23±0.65 | -7.849 | <0.001* |
| TG (mmol/L) | 1.62±0.54 | 0.87±0.25 | 9.419 | <0.001* |
| HDL-C (mmol/L) | 0.84±0.18 | 1.50±0.31 | -15.789 | <0.001* |
| LDL-C (mmol/L) | 2.25±0.45 | 1.97±0.59 | 3.159 | 0.002 |
| ApoA1 (g/L) | 0.87±0.25 | 1.36±0.28 | -10.614 | 0.000 |
| ApoB100 (g/L) | 0.98±0.33 | 0.77±0.18 | 4.104 | <0.001* |

Note: * indicates a P value less than 0.05. Abbreviation: C-reactive protein (CRP), syndecan-1 (SDC-1), white blood cells (WBC), platelets (PLT), interleukin 6 (IL-6), thrombin time (TT), activated partial thromboplastin time (APTT), fibrinogen (FIB), total cholesterol (TC), triglycerides (TG), high-density lipoprotein cholesterol (HDL-C), low density lipoprotein cholesterol (LDL-C), apolipoprotein A1 (ApoA1) and apolipoprotein B100 (ApoB100).

Table 2. Comparison of baseline clinical characteristics between refractory KD group and responsive KD group

| Clinical characteristics | Refractory KD (n= 31) | Responsive KD  (n=55) | χ2/t | P |
| --- | --- | --- | --- | --- |
| Male (%) | 20(51.61%) | 28(43.64%) | 1.488 | 0.223 |
| Age (years) | 3.36±1.65 | 3.33±1.57 | 0.105 | 0.917 |
| Weight (kg) | 15.44±3.17 | 15.22±3.11 | 0.871 | 0.756 |
| ALT(U/L) | 86.45±23.19 | 71.89±22.13 | -2.879 | 0.005 |
| AST(U/L) | 72.71±16.65 | 61.76±17.34 | -2.851 | 0.005 |
| SDC-1 (ng/mL) | 6.24±0.77 | 5.12±0.93 | 5.690 | 0.000 |
| CRP (mg/L) | 72.80±34.52 | 61.44±13.74 | 2.163 | 0.033 |
| IL-6 (pg/mL) | 74.12±13.53 | 70.09±8.90 | 1.665 | 0.100 |
| WBC (×109/L) | 16.48±2.29 | 15.35±2.19 | 2.278 | 0.025 |
| PLT (×109/L) | 363.48±56.85 | 349.96±59.75 | 1.025 | 0.308 |
| APTT (s) | 34.03±6.34 | 32.45±5.36 | 1.226 | 0.224 |
| TT (s) | 16.03±3.04 | 16.20±3.00 | -0.248 | 0.805 |
| FIB (g/L) | 2.65±1.52 | 2.46±0.54 | 0.859 | 0.393 |
| TC (mmol/L) | 3.31±0.66 | 3.39±0.59 | -0.639 | 0.525 |
| TG (mmol/L) | 1.86±0.61 | 1.48±0.44 | 3.316 | 0.001 |
| HDL-C (mmol/L) | 0.83±0.19 | 0.84±0.18 | -0.264 | 0.792 |
| LDL-C (mmol/L) | 2.27±0.42 | 2.23±0.47 | 0.377 | 0.707 |
| ApoA1 (g/L) | 0.87±0.25 | 0.88±0.25 | -0.185 | 0.853 |
| ApoB100 (g/L) | 1.05±0.39 | 0.93±0.29 | 1.643 | 0.104 |

Note: * indicates a P value less than 0.05. Abbreviation: C-reactive protein (CRP), syndecan-1 (SDC-1), white blood cells (WBC), platelets (PLT), interleukin 6 (IL-6), thrombin time (TT), activated partial thromboplastin time (APTT), fibrinogen (FIB), total cholesterol (TC), triglycerides (TG), high-density lipoprotein cholesterol (HDL-C), low density lipoprotein cholesterol (LDL-C), apolipoprotein A1 (ApoA1) and apolipoprotein B100 (ApoB100).

Table 3. Correlation analysis of general laboratory indicators and lipid indicators

|  | CRP (mg/L) | |  | SDC-1 | |  | PLT (×109/L) | |  | IL-6 (pg/mL) | |
| --- | --- | --- | --- | --- | --- | --- | --- | --- | --- | --- | --- |
|  | r | P |  | r | P |  | r | P |  | r | P |
| WBC(×109/L) | 0.135 | 0.217 |  | 0.035 | 0.749 |  | 0.085 | 0.438 |  | -0.032 | 0.773 |
| FIB(g/L) | 0.475 | 0.000* |  | 0.008 | 0.941 |  | 0.104 | 0.342 |  | 0.342 | 0.001* |
| APTT (s) | 0.150 | 0.168 |  | -0.039 | 0.722 |  | 0.018 | 0.873 |  | 0.216 | 0.045* |
| TT (s) | -0.097 | 0.372 |  | 0.035 | 0.751 |  | 0.080 | 0.466 |  | 0.009 | 0.932 |
| TC (mmol/L) | 0.001 | 0.990 |  | -0.047 | 0.664 |  | -0.008 | 0.944 |  | -0.155 | 0.155 |
| TG (mmol/L) | 0.223 | 0.039* |  | 0.250 | 0.020* |  | 0.002 | 0.987 |  | 0.491 | 0.000* |
| HDL-C (mmol/L) | 0.048 | 0.659 |  | -0.149 | 0.171 |  | 0.025 | 0.817 |  | 0.022 | 0.839 |
| LDL-C (mmol/L) | -0.243 | 0.024* |  | 0.000 | 0.993 |  | 0.227 | 0.035 |  | 0.004 | 0.972 |
| ApoA1 (g/L) | -0.265 | 0.014* |  | -0.106 | 0.330 |  | -0.047 | 0.670 |  | 0.171 | 0.115 |
| ApoB100 (g/L) | 0.404 | 0.000* |  | 0.223 | 0.039* |  | -0.097 | 0.374 |  | 0.253 | 0.019* |

Note: * indicates a P value less than 0.05. Abbreviation: C-reactive protein (CRP), syndecan-1 (SDC-1), platelets (PLT), interleukin 6 (IL-6), white blood cells (WBC), fibrinogen (FIB), activated partial thromboplastin time (APTT), thrombin time (TT), total cholesterol (TC), triglycerides (TG), high-density lipoprotein cholesterol (HDL-C), low density lipoprotein cholesterol (LDL-C), apolipoprotein A1 (ApoA1) and apolipoprotein B100 (ApoB100).

Table 4. SDC-1 levels in patients with different grades of CALs

|  | Mild CALs group (n=19) | Moderate CALs group (n=15) | Severe CALs group (n=7) | F | P |
| --- | --- | --- | --- | --- | --- |
| Coronary artery diameter (mm) | 4.19±2.80 | 6.57±3.18 | 9.30±2.55 | 8.468 | 0.001 |
| SDC-1 (ng/mL) | 5.52±0.87* | 6.18±0.54*# | 6.91±0.19# | 5.464 | 0.008 |

Note: * and # indicate a significant difference in the comparison. Abbreviation: syndecan-1 (SDC-1), coronary artery lesions (CALs).

Figure 3. ROC curve of SDC-1 diagnosis in CALs patients among KD patients. Abbreviation: Receiver operating characteristic (ROC), syndecan-1 (SDC-1), coronary artery lesions (CALs), Kawasaki disease (KD).

4.  Also, Status of treatment is not mentioned, either IVIG treated or not and the effect of treatment on SDC-1

Reply: Thank you for your attention to our research and for your valuable comments. The issue you raised regarding the treatment status (whether patients received IVIG therapy) and its impact on SDC-1 levels is indeed pertinent. The relevant content and information have been added into the article as suggested. However, the primary objective of our study was to explore the diagnostic value of SDC-1 in the identification of coronary artery lesions in KD patients and its correlation with multiple laboratory indicators, rather than assessing the effects of treatment on SDC-1 levels.

We concur that in future studies, particularly prospective ones, it is essential to meticulously record the treatment status of patients and to evaluate the potential impact of these treatments on SDC-1 levels. This will contribute to a more comprehensive understanding of the clinical significance of SDC-1 in KD patients.

Once again, we appreciate your suggestions and will take these considerations into account in our subsequent research endeavors, striving to provide more comprehensive data and analyses.

**Changs in the text:**

**1.5 Treatment Protocol**

All KD patients received IVIG treatment at a dose of 2 g/kg between days 4 and 10 of illness, in accordance with American Heart Association (AHA) guidelines. This was combined with medium- or high-dose aspirin (ASA) therapy. Additionally, patients with severe CALs underwent initial corticosteroid intensification therapy. These patients were treated with a modified RAISE protocol, which included 2 mg/kg/day of intravenous methylprednisolone (IVMP), administered twice daily, followed by a transition to oral prednisolone. Refractory KD cases also received a second IVIG treatment. (page 6, line 157-165)

5.  In addition, the status of KD based on treatment -either refractory or not -is not mentioned. I think that comparison of SDC-1 levels and either biomarker between refractory KD and responsive KD would be valuable in prognosis prior to treatment.

Reply: Thank you for the professional comment, which is highly appreciated. We strongly agree with your opinion. In the article, KD (n=86) was stratified into subgroups based on the degree of coronary artery injury: a group with no coronary artery injury (n=44), mild coronary artery lesions (mild CALs, n=18), moderate coronary artery lesions (moderate CALs, n=17), and severe coronary artery lesions (severe CALs, n=7). Within the original text, we have supplemented the analysis of these subgroups and additionally include a table differentiating between refractory KD and non-refractory KD.

**Changs in the text:**

**Methods**: 86 pediatric Kawasaki disease (KD) patients and 52 healthy controls admitted from January 2018 to December 2023 were retrospectively analyzed. Venous blood samples from KD patients were analyzed for white blood cells (WBC), platelets (PLT), C-reactive protein (CRP), interleukin-6 (IL-6), syndecan-1 (SDC-1), coagulation parameters, and lipid profiles. Correlations between these laboratory indicators were assessed. Receiver operating characteristic (ROC) curve analysis determined the diagnostic value of SDC-1 for coronary artery lesions (CALs) in KD patients. SDC-1 levels were further compared across different CAL severity groups.

**Results**: The levels of ALT, AST, WBC, PLT, CRP, IL-6, and SDC-1 in the KD group were significantly higher than those in the control group (P<0.05). Coagulation function analysis showed that APTT and TT were significantly increased, while FIB levels were significantly decreased in the KD group compared with the control group (P<0.05). Lipid profile analysis revealed that TC, HDL-C, and ApoA1 were significantly decreased, whereas TG, LDL-C, and ApoB100 were significantly increased in the KD group (P<0.05). Refractory KD patients exhibited significantly higher levels of ALT, AST, CRP, WBC, and TG compared to responsive KD patients (P<0.05). Correlation analysis indicated a strong positive correlation between PLT and LDL-C (r=0.813, P<0.001) and between IL-6 and TG (r=0.926, P<0.001), while CRP was negatively correlated with ApoA1 (r=-0.599, P=0.000). Among the 86 KD patients, 41 (47.67%) developed CALs, with 19 classified as mild, 15 as moderate, and 7 as severe. For predicting CALs among KD patients, the threshold of SDC-1 was identified as 5.5 ng/ml, with a sensitivity of 70.7%, specificity of 64.4%, positive predictive value of 65.91%, negative predictive value of 69.05%, and an AUC of 0.762 (95% confidence interval 0.662-0.861, P<0.001). SDC-1 levels significantly differed among the CAL severity groups (P<0.05), with higher levels observed in moderate compared to mild CALs, and in severe compared to moderate CALs (P<0.05). (page 2-3, line 35-61)

There were 86 subjects in the KD group, including 48 males (55.81%) and 38 females (44.19%). The age range was 3 months to 8.5 years, with a mean age of 3.34±1.59 years. There were 52 subjects in the control group, including 25 males (48.08%) and 27 females (51.92%). The age range was 4 months to 8.7 years, with a mean age of 3.48±1.60 years. (page 6-7, line 180-184)

Additionally, in liver function parameters, AST and ALT levels in the KD group were significantly higher than those in the control group (P<0.05). (Table 1). It is noteworthy that, compared with the refractory KD group, the levels of ALT, AST, CRP, WBC count and TG were significantly reduced in the responsive KD group (P<0.05). (Table 2) (page 7, line 191-195)

In the correlation analysis, we primarily examined the relationships between general laboratory indicators and lipid profiles. The results demonstrated a strong positive correlation between PLT and LDL-C (r=0.813, P<0.001) as well as IL-6 and TG (r=0.926, P<0.001). A moderate positive correlation was observed between SDC-1 and TG (r=0.485, P=0.034) and between IL-6 and ApoB100 (r=0.487, P=0.033). Additionally, there was a moderate negative correlation between CRP and ApoA1 (r=-0.487, P=0.033), with a stronger negative correlation observed between CRP and ApoA1 (r=-0.599, P=0.000). Moreover, a weaker correlation was found between IL-6 and TG (r=0.229, P=0.034) (Table 3) (page 7, line 198-206)

Among 86 KD patients in this study, 41 (47.67%) developed CALs. For predicting CALs among KD patients, the threshold of SDC-1 was identified as 5.5 ng/ml, with a sensitivity of 70.7%, specificity of 64.4%, positive predictive value of 65.91%, negative predictive value of 69.05%, and an AUC of 0.762 (95% confidence interval 0.662-0.861, P<0.001). The ROC curve of SDC-1 diagnosis in CALs patients among KD patients was presented in Figure 3. (page 7-8, line 208-213)

Of the 41 patients with CALs, 19 (46.34%) had mild CALs, 15 (36.59%) had moderate CALs, and 7 (17.07%) had severe CALs. Their mean coronary artery diameters were 4.19±2.80 mm, 6.57±3.18 mm, and 9.30±2.55 mm (page 8, line 215-217)

Table 1. Comparison of baseline clinical characteristics between KD group and control group

| Clinical characteristics | KD group (n=86) | Control group (n=52) | χ2/t | P |
| --- | --- | --- | --- | --- |
| Male (%) | 48（55.82%） | 25（48.08%） | 0.779 | 0.378 |
| Age (years) | 3.34±1.59 | 3.48±1.60 | -0.489 | 0.625 |
| Weight (kg) | 15.30±3.11 | 15.23±3.07 | 0.125 | 0.901 |
| ALT(U/L) | 77.14±23.46 | 26.35±10.37 | 14.748 | 0.000 |
| AST(U/L) | 65.71±17.80 | 28.81±8.73 | 13.957 | 0.000 |
| SDC-1 (ng/mL) | 5.52±1.02 | 2.51±0.65 | 19.030 | <0.001* |
| CRP (mg/L) | 62.53±23.88 | 5.69±1.60 | 18.012 | <0.001* |
| IL-6 (pg/mL) | 71.54±10.90 | 5.02±2.10 | 43.479 | <0.001* |
| WBC (×109/L) | 15.76±2.28 | 6.57±0.88 | 27.830 | <0.001* |
| PLT (×109/L) | 354.84±58.75 | 244.31±38.90 | 12.054 | <0.001* |
| APTT (s) | 33.02±5.75 | 21.23±1.64 | 14.430 | <0.001* |
| TT (s) | 16.14±2.99 | 14.40±2.65 | 3.442 | 0.001 |
| FIB (g/L) | 2.53±1.01 | 4.91±1.43 | -11.455 | <0.001* |
| TC (mmol/L) | 3.36±0.61 | 4.23±0.65 | -7.849 | <0.001* |
| TG (mmol/L) | 1.62±0.54 | 0.87±0.25 | 9.419 | <0.001* |
| HDL-C (mmol/L) | 0.84±0.18 | 1.50±0.31 | -15.789 | <0.001* |
| LDL-C (mmol/L) | 2.25±0.45 | 1.97±0.59 | 3.159 | 0.002 |
| ApoA1 (g/L) | 0.87±0.25 | 1.36±0.28 | -10.614 | 0.000 |
| ApoB100 (g/L) | 0.98±0.33 | 0.77±0.18 | 4.104 | <0.001* |

Note: * indicates a P value less than 0.05. Abbreviation: C-reactive protein (CRP), syndecan-1 (SDC-1), white blood cells (WBC), platelets (PLT), interleukin 6 (IL-6), thrombin time (TT), activated partial thromboplastin time (APTT), fibrinogen (FIB), total cholesterol (TC), triglycerides (TG), high-density lipoprotein cholesterol (HDL-C), low density lipoprotein cholesterol (LDL-C), apolipoprotein A1 (ApoA1) and apolipoprotein B100 (ApoB100).

Table 2. Comparison of baseline clinical characteristics between refractory KD group and responsive KD group

| Clinical characteristics | Refractory KD (n= 31) | Responsive KD  (n=55) | χ2/t | P |
| --- | --- | --- | --- | --- |
| Male (%) | 20(51.61%) | 28(43.64%) | 1.488 | 0.223 |
| Age (years) | 3.36±1.65 | 3.33±1.57 | 0.105 | 0.917 |
| Weight (kg) | 15.44±3.17 | 15.22±3.11 | 0.871 | 0.756 |
| ALT(U/L) | 86.45±23.19 | 71.89±22.13 | -2.879 | 0.005 |
| AST(U/L) | 72.71±16.65 | 61.76±17.34 | -2.851 | 0.005 |
| SDC-1 (ng/mL) | 6.24±0.77 | 5.12±0.93 | 5.690 | 0.000 |
| CRP (mg/L) | 72.80±34.52 | 61.44±13.74 | 2.163 | 0.033 |
| IL-6 (pg/mL) | 74.12±13.53 | 70.09±8.90 | 1.665 | 0.100 |
| WBC (×109/L) | 16.48±2.29 | 15.35±2.19 | 2.278 | 0.025 |
| PLT (×109/L) | 363.48±56.85 | 349.96±59.75 | 1.025 | 0.308 |
| APTT (s) | 34.03±6.34 | 32.45±5.36 | 1.226 | 0.224 |
| TT (s) | 16.03±3.04 | 16.20±3.00 | -0.248 | 0.805 |
| FIB (g/L) | 2.65±1.52 | 2.46±0.54 | 0.859 | 0.393 |
| TC (mmol/L) | 3.31±0.66 | 3.39±0.59 | -0.639 | 0.525 |
| TG (mmol/L) | 1.86±0.61 | 1.48±0.44 | 3.316 | 0.001 |
| HDL-C (mmol/L) | 0.83±0.19 | 0.84±0.18 | -0.264 | 0.792 |
| LDL-C (mmol/L) | 2.27±0.42 | 2.23±0.47 | 0.377 | 0.707 |
| ApoA1 (g/L) | 0.87±0.25 | 0.88±0.25 | -0.185 | 0.853 |
| ApoB100 (g/L) | 1.05±0.39 | 0.93±0.29 | 1.643 | 0.104 |

Note: * indicates a P value less than 0.05. Abbreviation: C-reactive protein (CRP), syndecan-1 (SDC-1), white blood cells (WBC), platelets (PLT), interleukin 6 (IL-6), thrombin time (TT), activated partial thromboplastin time (APTT), fibrinogen (FIB), total cholesterol (TC), triglycerides (TG), high-density lipoprotein cholesterol (HDL-C), low density lipoprotein cholesterol (LDL-C), apolipoprotein A1 (ApoA1) and apolipoprotein B100 (ApoB100).

Table 3. Correlation analysis of general laboratory indicators and lipid indicators

|  | CRP (mg/L) | |  | SDC-1 | |  | PLT (×109/L) | |  | IL-6 (pg/mL) | |
| --- | --- | --- | --- | --- | --- | --- | --- | --- | --- | --- | --- |
|  | r | P |  | r | P |  | r | P |  | r | P |
| WBC(×109/L) | 0.135 | 0.217 |  | 0.035 | 0.749 |  | 0.085 | 0.438 |  | -0.032 | 0.773 |
| FIB(g/L) | 0.475 | 0.000* |  | 0.008 | 0.941 |  | 0.104 | 0.342 |  | 0.342 | 0.001* |
| APTT (s) | 0.150 | 0.168 |  | -0.039 | 0.722 |  | 0.018 | 0.873 |  | 0.216 | 0.045* |
| TT (s) | -0.097 | 0.372 |  | 0.035 | 0.751 |  | 0.080 | 0.466 |  | 0.009 | 0.932 |
| TC (mmol/L) | 0.001 | 0.990 |  | -0.047 | 0.664 |  | -0.008 | 0.944 |  | -0.155 | 0.155 |
| TG (mmol/L) | 0.223 | 0.039* |  | 0.250 | 0.020* |  | 0.002 | 0.987 |  | 0.491 | 0.000* |
| HDL-C (mmol/L) | 0.048 | 0.659 |  | -0.149 | 0.171 |  | 0.025 | 0.817 |  | 0.022 | 0.839 |
| LDL-C (mmol/L) | -0.243 | 0.024* |  | 0.000 | 0.993 |  | 0.227 | 0.035 |  | 0.004 | 0.972 |
| ApoA1 (g/L) | -0.265 | 0.014* |  | -0.106 | 0.330 |  | -0.047 | 0.670 |  | 0.171 | 0.115 |
| ApoB100 (g/L) | 0.404 | 0.000* |  | 0.223 | 0.039* |  | -0.097 | 0.374 |  | 0.253 | 0.019* |

Note: * indicates a P value less than 0.05. Abbreviation: C-reactive protein (CRP), syndecan-1 (SDC-1), platelets (PLT), interleukin 6 (IL-6), white blood cells (WBC), fibrinogen (FIB), activated partial thromboplastin time (APTT), thrombin time (TT), total cholesterol (TC), triglycerides (TG), high-density lipoprotein cholesterol (HDL-C), low density lipoprotein cholesterol (LDL-C), apolipoprotein A1 (ApoA1) and apolipoprotein B100 (ApoB100).

Table 4. SDC-1 levels in patients with different grades of CALs

|  | Mild CALs group (n=19) | Moderate CALs group (n=15) | Severe CALs group (n=7) | F | P |
| --- | --- | --- | --- | --- | --- |
| Coronary artery diameter (mm) | 4.19±2.80 | 6.57±3.18 | 9.30±2.55 | 8.468 | 0.001 |
| SDC-1 (ng/mL) | 5.52±0.87* | 6.18±0.54*# | 6.91±0.19# | 5.464 | 0.008 |

Note: * and # indicate a significant difference in the comparison. Abbreviation: syndecan-1 (SDC-1), coronary artery lesions (CALs).

Figure 3. ROC curve of SDC-1 diagnosis in CALs patients among KD patients. Abbreviation: Receiver operating characteristic (ROC), syndecan-1 (SDC-1), coronary artery lesions (CALs), Kawasaki disease (KD).

6.  Why only typical KD patients were added, in our practice, Atypical KD are arising so studying levels of SDC-1 and other biomarkers in atypical KD and comparing with typical patients would help to explain the pattern of presentations.

Reply: Thank you for the professional comment, which is highly appreciated. We have categorized the additional cases into refractory KD and non-refractory KD, and have statistically analyzed the biomarker levels within these subgroups. Due to considerations of resources and research funding, we will consider including the atypical KD subgroup in subsequent studies.

**Changs in the text:**

**Methods**: 86 pediatric Kawasaki disease (KD) patients and 52 healthy controls admitted from January 2018 to December 2023 were retrospectively analyzed. Venous blood samples from KD patients were analyzed for white blood cells (WBC), platelets (PLT), C-reactive protein (CRP), interleukin-6 (IL-6), syndecan-1 (SDC-1), coagulation parameters, and lipid profiles. Correlations between these laboratory indicators were assessed. Receiver operating characteristic (ROC) curve analysis determined the diagnostic value of SDC-1 for coronary artery lesions (CALs) in KD patients. SDC-1 levels were further compared across different CAL severity groups.

**Results**: The levels of ALT, AST, WBC, PLT, CRP, IL-6, and SDC-1 in the KD group were significantly higher than those in the control group (P<0.05). Coagulation function analysis showed that APTT and TT were significantly increased, while FIB levels were significantly decreased in the KD group compared with the control group (P<0.05). Lipid profile analysis revealed that TC, HDL-C, and ApoA1 were significantly decreased, whereas TG, LDL-C, and ApoB100 were significantly increased in the KD group (P<0.05). Refractory KD patients exhibited significantly higher levels of ALT, AST, CRP, WBC, and TG compared to responsive KD patients (P<0.05). Correlation analysis indicated a strong positive correlation between PLT and LDL-C (r=0.813, P<0.001) and between IL-6 and TG (r=0.926, P<0.001), while CRP was negatively correlated with ApoA1 (r=-0.599, P=0.000). Among the 86 KD patients, 41 (47.67%) developed CALs, with 19 classified as mild, 15 as moderate, and 7 as severe. For predicting CALs among KD patients, the threshold of SDC-1 was identified as 5.5 ng/ml, with a sensitivity of 70.7%, specificity of 64.4%, positive predictive value of 65.91%, negative predictive value of 69.05%, and an AUC of 0.762 (95% confidence interval 0.662-0.861, P<0.001). SDC-1 levels significantly differed among the CAL severity groups (P<0.05), with higher levels observed in moderate compared to mild CALs, and in severe compared to moderate CALs (P<0.05). (page 2-3, line 35-61)

There were 86 subjects in the KD group, including 48 males (55.81%) and 38 females (44.19%). The age range was 3 months to 8.5 years, with a mean age of 3.34±1.59 years. There were 52 subjects in the control group, including 25 males (48.08%) and 27 females (51.92%). The age range was 4 months to 8.7 years, with a mean age of 3.48±1.60 years. (page 6-7, line 180-184)

Additionally, in liver function parameters, AST and ALT levels in the KD group were significantly higher than those in the control group (P<0.05). (Table 1). It is noteworthy that, compared with the refractory KD group, the levels of ALT, AST, CRP, WBC count and TG were significantly reduced in the responsive KD group (P<0.05). (Table 2) (page 7, line 191-195)

In the correlation analysis, we primarily examined the relationships between general laboratory indicators and lipid profiles. The results demonstrated a strong positive correlation between PLT and LDL-C (r=0.813, P<0.001) as well as IL-6 and TG (r=0.926, P<0.001). A moderate positive correlation was observed between SDC-1 and TG (r=0.485, P=0.034) and between IL-6 and ApoB100 (r=0.487, P=0.033). Additionally, there was a moderate negative correlation between CRP and ApoA1 (r=-0.487, P=0.033), with a stronger negative correlation observed between CRP and ApoA1 (r=-0.599, P=0.000). Moreover, a weaker correlation was found between IL-6 and TG (r=0.229, P=0.034) (Table 3) (page 7, line 198-206)

Among 86 KD patients in this study, 41 (47.67%) developed CALs. For predicting CALs among KD patients, the threshold of SDC-1 was identified as 5.5 ng/ml, with a sensitivity of 70.7%, specificity of 64.4%, positive predictive value of 65.91%, negative predictive value of 69.05%, and an AUC of 0.762 (95% confidence interval 0.662-0.861, P<0.001). The ROC curve of SDC-1 diagnosis in CALs patients among KD patients was presented in Figure 3. (page 7-8, line 208-213)

Of the 41 patients with CALs, 19 (46.34%) had mild CALs, 15 (36.59%) had moderate CALs, and 7 (17.07%) had severe CALs. Their mean coronary artery diameters were 4.19±2.80 mm, 6.57±3.18 mm, and 9.30±2.55 mm (page 8, line 215-217)

Table 1. Comparison of baseline clinical characteristics between KD group and control group

| Clinical characteristics | KD group (n=86) | Control group (n=52) | χ2/t | P |
| --- | --- | --- | --- | --- |
| Male (%) | 48（55.82%） | 25（48.08%） | 0.779 | 0.378 |
| Age (years) | 3.34±1.59 | 3.48±1.60 | -0.489 | 0.625 |
| Weight (kg) | 15.30±3.11 | 15.23±3.07 | 0.125 | 0.901 |
| ALT(U/L) | 77.14±23.46 | 26.35±10.37 | 14.748 | 0.000 |
| AST(U/L) | 65.71±17.80 | 28.81±8.73 | 13.957 | 0.000 |
| SDC-1 (ng/mL) | 5.52±1.02 | 2.51±0.65 | 19.030 | <0.001* |
| CRP (mg/L) | 62.53±23.88 | 5.69±1.60 | 18.012 | <0.001* |
| IL-6 (pg/mL) | 71.54±10.90 | 5.02±2.10 | 43.479 | <0.001* |
| WBC (×109/L) | 15.76±2.28 | 6.57±0.88 | 27.830 | <0.001* |
| PLT (×109/L) | 354.84±58.75 | 244.31±38.90 | 12.054 | <0.001* |
| APTT (s) | 33.02±5.75 | 21.23±1.64 | 14.430 | <0.001* |
| TT (s) | 16.14±2.99 | 14.40±2.65 | 3.442 | 0.001 |
| FIB (g/L) | 2.53±1.01 | 4.91±1.43 | -11.455 | <0.001* |
| TC (mmol/L) | 3.36±0.61 | 4.23±0.65 | -7.849 | <0.001* |
| TG (mmol/L) | 1.62±0.54 | 0.87±0.25 | 9.419 | <0.001* |
| HDL-C (mmol/L) | 0.84±0.18 | 1.50±0.31 | -15.789 | <0.001* |
| LDL-C (mmol/L) | 2.25±0.45 | 1.97±0.59 | 3.159 | 0.002 |
| ApoA1 (g/L) | 0.87±0.25 | 1.36±0.28 | -10.614 | 0.000 |
| ApoB100 (g/L) | 0.98±0.33 | 0.77±0.18 | 4.104 | <0.001* |

Note: * indicates a P value less than 0.05. Abbreviation: C-reactive protein (CRP), syndecan-1 (SDC-1), white blood cells (WBC), platelets (PLT), interleukin 6 (IL-6), thrombin time (TT), activated partial thromboplastin time (APTT), fibrinogen (FIB), total cholesterol (TC), triglycerides (TG), high-density lipoprotein cholesterol (HDL-C), low density lipoprotein cholesterol (LDL-C), apolipoprotein A1 (ApoA1) and apolipoprotein B100 (ApoB100).

Table 2. Comparison of baseline clinical characteristics between refractory KD group and responsive KD group

| Clinical characteristics | Refractory KD (n= 31) | Responsive KD  (n=55) | χ2/t | P |
| --- | --- | --- | --- | --- |
| Male (%) | 20(51.61%) | 28(43.64%) | 1.488 | 0.223 |
| Age (years) | 3.36±1.65 | 3.33±1.57 | 0.105 | 0.917 |
| Weight (kg) | 15.44±3.17 | 15.22±3.11 | 0.871 | 0.756 |
| ALT(U/L) | 86.45±23.19 | 71.89±22.13 | -2.879 | 0.005 |
| AST(U/L) | 72.71±16.65 | 61.76±17.34 | -2.851 | 0.005 |
| SDC-1 (ng/mL) | 6.24±0.77 | 5.12±0.93 | 5.690 | 0.000 |
| CRP (mg/L) | 72.80±34.52 | 61.44±13.74 | 2.163 | 0.033 |
| IL-6 (pg/mL) | 74.12±13.53 | 70.09±8.90 | 1.665 | 0.100 |
| WBC (×109/L) | 16.48±2.29 | 15.35±2.19 | 2.278 | 0.025 |
| PLT (×109/L) | 363.48±56.85 | 349.96±59.75 | 1.025 | 0.308 |
| APTT (s) | 34.03±6.34 | 32.45±5.36 | 1.226 | 0.224 |
| TT (s) | 16.03±3.04 | 16.20±3.00 | -0.248 | 0.805 |
| FIB (g/L) | 2.65±1.52 | 2.46±0.54 | 0.859 | 0.393 |
| TC (mmol/L) | 3.31±0.66 | 3.39±0.59 | -0.639 | 0.525 |
| TG (mmol/L) | 1.86±0.61 | 1.48±0.44 | 3.316 | 0.001 |
| HDL-C (mmol/L) | 0.83±0.19 | 0.84±0.18 | -0.264 | 0.792 |
| LDL-C (mmol/L) | 2.27±0.42 | 2.23±0.47 | 0.377 | 0.707 |
| ApoA1 (g/L) | 0.87±0.25 | 0.88±0.25 | -0.185 | 0.853 |
| ApoB100 (g/L) | 1.05±0.39 | 0.93±0.29 | 1.643 | 0.104 |

Note: * indicates a P value less than 0.05. Abbreviation: C-reactive protein (CRP), syndecan-1 (SDC-1), white blood cells (WBC), platelets (PLT), interleukin 6 (IL-6), thrombin time (TT), activated partial thromboplastin time (APTT), fibrinogen (FIB), total cholesterol (TC), triglycerides (TG), high-density lipoprotein cholesterol (HDL-C), low density lipoprotein cholesterol (LDL-C), apolipoprotein A1 (ApoA1) and apolipoprotein B100 (ApoB100).

Table 3. Correlation analysis of general laboratory indicators and lipid indicators

|  | CRP (mg/L) | |  | SDC-1 | |  | PLT (×109/L) | |  | IL-6 (pg/mL) | |
| --- | --- | --- | --- | --- | --- | --- | --- | --- | --- | --- | --- |
|  | r | P |  | r | P |  | r | P |  | r | P |
| WBC(×109/L) | 0.135 | 0.217 |  | 0.035 | 0.749 |  | 0.085 | 0.438 |  | -0.032 | 0.773 |
| FIB(g/L) | 0.475 | 0.000* |  | 0.008 | 0.941 |  | 0.104 | 0.342 |  | 0.342 | 0.001* |
| APTT (s) | 0.150 | 0.168 |  | -0.039 | 0.722 |  | 0.018 | 0.873 |  | 0.216 | 0.045* |
| TT (s) | -0.097 | 0.372 |  | 0.035 | 0.751 |  | 0.080 | 0.466 |  | 0.009 | 0.932 |
| TC (mmol/L) | 0.001 | 0.990 |  | -0.047 | 0.664 |  | -0.008 | 0.944 |  | -0.155 | 0.155 |
| TG (mmol/L) | 0.223 | 0.039* |  | 0.250 | 0.020* |  | 0.002 | 0.987 |  | 0.491 | 0.000* |
| HDL-C (mmol/L) | 0.048 | 0.659 |  | -0.149 | 0.171 |  | 0.025 | 0.817 |  | 0.022 | 0.839 |
| LDL-C (mmol/L) | -0.243 | 0.024* |  | 0.000 | 0.993 |  | 0.227 | 0.035 |  | 0.004 | 0.972 |
| ApoA1 (g/L) | -0.265 | 0.014* |  | -0.106 | 0.330 |  | -0.047 | 0.670 |  | 0.171 | 0.115 |
| ApoB100 (g/L) | 0.404 | 0.000* |  | 0.223 | 0.039* |  | -0.097 | 0.374 |  | 0.253 | 0.019* |

Note: * indicates a P value less than 0.05. Abbreviation: C-reactive protein (CRP), syndecan-1 (SDC-1), platelets (PLT), interleukin 6 (IL-6), white blood cells (WBC), fibrinogen (FIB), activated partial thromboplastin time (APTT), thrombin time (TT), total cholesterol (TC), triglycerides (TG), high-density lipoprotein cholesterol (HDL-C), low density lipoprotein cholesterol (LDL-C), apolipoprotein A1 (ApoA1) and apolipoprotein B100 (ApoB100).

Table 4. SDC-1 levels in patients with different grades of CALs

|  | Mild CALs group (n=19) | Moderate CALs group (n=15) | Severe CALs group (n=7) | F | P |
| --- | --- | --- | --- | --- | --- |
| Coronary artery diameter (mm) | 4.19±2.80 | 6.57±3.18 | 9.30±2.55 | 8.468 | 0.001 |
| SDC-1 (ng/mL) | 5.52±0.87* | 6.18±0.54*# | 6.91±0.19# | 5.464 | 0.008 |

Note: * and # indicate a significant difference in the comparison. Abbreviation: syndecan-1 (SDC-1), coronary artery lesions (CALs).

Figure 3. ROC curve of SDC-1 diagnosis in CALs patients among KD patients. Abbreviation: Receiver operating characteristic (ROC), syndecan-1 (SDC-1), coronary artery lesions (CALs), Kawasaki disease (KD).

7.  Authors should mention methods of coronary assessment( Echocardiography, CT) machine details.

Reply: Thank you for the valuable suggestion, which help improve the accuracy of the manuscript. CALs in this study were evaluated using echocardiography. The imaging was performed with a [GE Vivid E95, manufactured by GE Healthcare], which was utilized to obtain measurements of coronary artery dimensions. The relevant information has been added into the text as suggested.

**Changs in the text:**

CALs in this study were evaluated using echocardiography. The imaging was performed with a [GE Vivid E95, manufactured by GE Healthcare], which was utilized to obtain measurements of coronary artery dimensions. (page 5, line 143-145)

¶Results
This section provides comprehensive overview of the baseline clinical characteristics and the correlation analysis between various laboratory indicators and lipid profiles in the study cohort. However, there are a few weak points that could be addressed.
1.  Sample size:
*  The sample size for the control group (n=32) is significantly smaller compared to the KD group (n=65).
*  This disparity in sample size could impact the statistical power and the reliability of the comparisons between the two groups

Reply: Thnak you for your attention to our study and the insightful comments you have provided. The issue of the smaller sample size in the control group, which you have highlighted, is indeed an important aspect that we considered in our study design.

We acknowledge that the sample size of the control group is relatively small, primarily due to the challenges in recruiting healthy children, especially when blood tests are involved. Despite these difficulties, we endeavored to recruit an adequate number of control subjects within the constraints of our resources and timeframe to ensure the feasibility of our research. We have already enrolled patients and supplemented the sample size in the revised manuscript.

We concur that the disparity in sample sizes may impact the statistical power and the reliability of the comparisons between the two groups. In our analysis, we employed appropriate statistical tests (such as independent samples t-tests, analysis of variance, etc.) and made necessary data transformations and model adjustments to mitigate the impact of the sample size difference.

To ensure the reliability of our results, we paid particular attention to the influence of sample size in our analysis. We utilized the Kolmogorov-Smirnov test to assess the normality of the data distribution and selected appropriate statistical methods based on the characteristics of the data distribution. Furthermore, we calculated effect sizes and confidence intervals for a more comprehensive assessment of the significance of our findings.

We believe that by increasing the sample size, we can more accurately assess the performance of SDC-1 and other biomarkers in KD patients, thereby providing stronger support for clinical practice. The relevant content has been mentioned in the limitations.

Once again, we thank you for your valuable comments. We look forward to any further guidance and suggestions you may have.

**Changs in the text:**

**Methods**: 86 pediatric Kawasaki disease (KD) patients and 52 healthy controls admitted from January 2018 to December 2023 were retrospectively analyzed. Venous blood samples from KD patients were analyzed for white blood cells (WBC), platelets (PLT), C-reactive protein (CRP), interleukin-6 (IL-6), syndecan-1 (SDC-1), coagulation parameters, and lipid profiles. Correlations between these laboratory indicators were assessed. Receiver operating characteristic (ROC) curve analysis determined the diagnostic value of SDC-1 for coronary artery lesions (CALs) in KD patients. SDC-1 levels were further compared across different CAL severity groups.

**Results**: The levels of ALT, AST, WBC, PLT, CRP, IL-6, and SDC-1 in the KD group were significantly higher than those in the control group (P<0.05). Coagulation function analysis showed that APTT and TT were significantly increased, while FIB levels were significantly decreased in the KD group compared with the control group (P<0.05). Lipid profile analysis revealed that TC, HDL-C, and ApoA1 were significantly decreased, whereas TG, LDL-C, and ApoB100 were significantly increased in the KD group (P<0.05). Refractory KD patients exhibited significantly higher levels of ALT, AST, CRP, WBC, and TG compared to responsive KD patients (P<0.05). Correlation analysis indicated a strong positive correlation between PLT and LDL-C (r=0.813, P<0.001) and between IL-6 and TG (r=0.926, P<0.001), while CRP was negatively correlated with ApoA1 (r=-0.599, P=0.000). Among the 86 KD patients, 41 (47.67%) developed CALs, with 19 classified as mild, 15 as moderate, and 7 as severe. For predicting CALs among KD patients, the threshold of SDC-1 was identified as 5.5 ng/ml, with a sensitivity of 70.7%, specificity of 64.4%, positive predictive value of 65.91%, negative predictive value of 69.05%, and an AUC of 0.762 (95% confidence interval 0.662-0.861, P<0.001). SDC-1 levels significantly differed among the CAL severity groups (P<0.05), with higher levels observed in moderate compared to mild CALs, and in severe compared to moderate CALs (P<0.05). (page 2-3, line 35-61)

There were 86 subjects in the KD group, including 48 males (55.81%) and 38 females (44.19%). The age range was 3 months to 8.5 years, with a mean age of 3.34±1.59 years. There were 52 subjects in the control group, including 25 males (48.08%) and 27 females (51.92%). The age range was 4 months to 8.7 years, with a mean age of 3.48±1.60 years. (page 6-7, line 180-184)

Additionally, in liver function parameters, AST and ALT levels in the KD group were significantly higher than those in the control group (P<0.05). (Table 1). It is noteworthy that, compared with the refractory KD group, the levels of ALT, AST, CRP, WBC count and TG were significantly reduced in the responsive KD group (P<0.05). (Table 2) (page 7, line 191-195)

In the correlation analysis, we primarily examined the relationships between general laboratory indicators and lipid profiles. The results demonstrated a strong positive correlation between PLT and LDL-C (r=0.813, P<0.001) as well as IL-6 and TG (r=0.926, P<0.001). A moderate positive correlation was observed between SDC-1 and TG (r=0.485, P=0.034) and between IL-6 and ApoB100 (r=0.487, P=0.033). Additionally, there was a moderate negative correlation between CRP and ApoA1 (r=-0.487, P=0.033), with a stronger negative correlation observed between CRP and ApoA1 (r=-0.599, P=0.000). Moreover, a weaker correlation was found between IL-6 and TG (r=0.229, P=0.034) (Table 3) (page 7, line 198-206)

Among 86 KD patients in this study, 41 (47.67%) developed CALs. For predicting CALs among KD patients, the threshold of SDC-1 was identified as 5.5 ng/ml, with a sensitivity of 70.7%, specificity of 64.4%, positive predictive value of 65.91%, negative predictive value of 69.05%, and an AUC of 0.762 (95% confidence interval 0.662-0.861, P<0.001). The ROC curve of SDC-1 diagnosis in CALs patients among KD patients was presented in Figure 3. (page 7-8, line 208-213)

Of the 41 patients with CALs, 19 (46.34%) had mild CALs, 15 (36.59%) had moderate CALs, and 7 (17.07%) had severe CALs. Their mean coronary artery diameters were 4.19±2.80 mm, 6.57±3.18 mm, and 9.30±2.55 mm (page 8, line 215-217)

Table 1. Comparison of baseline clinical characteristics between KD group and control group

| Clinical characteristics | KD group (n=86) | Control group (n=52) | χ2/t | P |
| --- | --- | --- | --- | --- |
| Male (%) | 48（55.82%） | 25（48.08%） | 0.779 | 0.378 |
| Age (years) | 3.34±1.59 | 3.48±1.60 | -0.489 | 0.625 |
| Weight (kg) | 15.30±3.11 | 15.23±3.07 | 0.125 | 0.901 |
| ALT(U/L) | 77.14±23.46 | 26.35±10.37 | 14.748 | 0.000 |
| AST(U/L) | 65.71±17.80 | 28.81±8.73 | 13.957 | 0.000 |
| SDC-1 (ng/mL) | 5.52±1.02 | 2.51±0.65 | 19.030 | <0.001* |
| CRP (mg/L) | 62.53±23.88 | 5.69±1.60 | 18.012 | <0.001* |
| IL-6 (pg/mL) | 71.54±10.90 | 5.02±2.10 | 43.479 | <0.001* |
| WBC (×109/L) | 15.76±2.28 | 6.57±0.88 | 27.830 | <0.001* |
| PLT (×109/L) | 354.84±58.75 | 244.31±38.90 | 12.054 | <0.001* |
| APTT (s) | 33.02±5.75 | 21.23±1.64 | 14.430 | <0.001* |
| TT (s) | 16.14±2.99 | 14.40±2.65 | 3.442 | 0.001 |
| FIB (g/L) | 2.53±1.01 | 4.91±1.43 | -11.455 | <0.001* |
| TC (mmol/L) | 3.36±0.61 | 4.23±0.65 | -7.849 | <0.001* |
| TG (mmol/L) | 1.62±0.54 | 0.87±0.25 | 9.419 | <0.001* |
| HDL-C (mmol/L) | 0.84±0.18 | 1.50±0.31 | -15.789 | <0.001* |
| LDL-C (mmol/L) | 2.25±0.45 | 1.97±0.59 | 3.159 | 0.002 |
| ApoA1 (g/L) | 0.87±0.25 | 1.36±0.28 | -10.614 | 0.000 |
| ApoB100 (g/L) | 0.98±0.33 | 0.77±0.18 | 4.104 | <0.001* |

Note: * indicates a P value less than 0.05. Abbreviation: C-reactive protein (CRP), syndecan-1 (SDC-1), white blood cells (WBC), platelets (PLT), interleukin 6 (IL-6), thrombin time (TT), activated partial thromboplastin time (APTT), fibrinogen (FIB), total cholesterol (TC), triglycerides (TG), high-density lipoprotein cholesterol (HDL-C), low density lipoprotein cholesterol (LDL-C), apolipoprotein A1 (ApoA1) and apolipoprotein B100 (ApoB100).

Table 2. Comparison of baseline clinical characteristics between refractory KD group and responsive KD group

| Clinical characteristics | Refractory KD (n= 31) | Responsive KD  (n=55) | χ2/t | P |
| --- | --- | --- | --- | --- |
| Male (%) | 20(51.61%) | 28(43.64%) | 1.488 | 0.223 |
| Age (years) | 3.36±1.65 | 3.33±1.57 | 0.105 | 0.917 |
| Weight (kg) | 15.44±3.17 | 15.22±3.11 | 0.871 | 0.756 |
| ALT(U/L) | 86.45±23.19 | 71.89±22.13 | -2.879 | 0.005 |
| AST(U/L) | 72.71±16.65 | 61.76±17.34 | -2.851 | 0.005 |
| SDC-1 (ng/mL) | 6.24±0.77 | 5.12±0.93 | 5.690 | 0.000 |
| CRP (mg/L) | 72.80±34.52 | 61.44±13.74 | 2.163 | 0.033 |
| IL-6 (pg/mL) | 74.12±13.53 | 70.09±8.90 | 1.665 | 0.100 |
| WBC (×109/L) | 16.48±2.29 | 15.35±2.19 | 2.278 | 0.025 |
| PLT (×109/L) | 363.48±56.85 | 349.96±59.75 | 1.025 | 0.308 |
| APTT (s) | 34.03±6.34 | 32.45±5.36 | 1.226 | 0.224 |
| TT (s) | 16.03±3.04 | 16.20±3.00 | -0.248 | 0.805 |
| FIB (g/L) | 2.65±1.52 | 2.46±0.54 | 0.859 | 0.393 |
| TC (mmol/L) | 3.31±0.66 | 3.39±0.59 | -0.639 | 0.525 |
| TG (mmol/L) | 1.86±0.61 | 1.48±0.44 | 3.316 | 0.001 |
| HDL-C (mmol/L) | 0.83±0.19 | 0.84±0.18 | -0.264 | 0.792 |
| LDL-C (mmol/L) | 2.27±0.42 | 2.23±0.47 | 0.377 | 0.707 |
| ApoA1 (g/L) | 0.87±0.25 | 0.88±0.25 | -0.185 | 0.853 |
| ApoB100 (g/L) | 1.05±0.39 | 0.93±0.29 | 1.643 | 0.104 |

Note: * indicates a P value less than 0.05. Abbreviation: C-reactive protein (CRP), syndecan-1 (SDC-1), white blood cells (WBC), platelets (PLT), interleukin 6 (IL-6), thrombin time (TT), activated partial thromboplastin time (APTT), fibrinogen (FIB), total cholesterol (TC), triglycerides (TG), high-density lipoprotein cholesterol (HDL-C), low density lipoprotein cholesterol (LDL-C), apolipoprotein A1 (ApoA1) and apolipoprotein B100 (ApoB100).

Table 3. Correlation analysis of general laboratory indicators and lipid indicators

|  | CRP (mg/L) | |  | SDC-1 | |  | PLT (×109/L) | |  | IL-6 (pg/mL) | |
| --- | --- | --- | --- | --- | --- | --- | --- | --- | --- | --- | --- |
|  | r | P |  | r | P |  | r | P |  | r | P |
| WBC(×109/L) | 0.135 | 0.217 |  | 0.035 | 0.749 |  | 0.085 | 0.438 |  | -0.032 | 0.773 |
| FIB(g/L) | 0.475 | 0.000* |  | 0.008 | 0.941 |  | 0.104 | 0.342 |  | 0.342 | 0.001* |
| APTT (s) | 0.150 | 0.168 |  | -0.039 | 0.722 |  | 0.018 | 0.873 |  | 0.216 | 0.045* |
| TT (s) | -0.097 | 0.372 |  | 0.035 | 0.751 |  | 0.080 | 0.466 |  | 0.009 | 0.932 |
| TC (mmol/L) | 0.001 | 0.990 |  | -0.047 | 0.664 |  | -0.008 | 0.944 |  | -0.155 | 0.155 |
| TG (mmol/L) | 0.223 | 0.039* |  | 0.250 | 0.020* |  | 0.002 | 0.987 |  | 0.491 | 0.000* |
| HDL-C (mmol/L) | 0.048 | 0.659 |  | -0.149 | 0.171 |  | 0.025 | 0.817 |  | 0.022 | 0.839 |
| LDL-C (mmol/L) | -0.243 | 0.024* |  | 0.000 | 0.993 |  | 0.227 | 0.035 |  | 0.004 | 0.972 |
| ApoA1 (g/L) | -0.265 | 0.014* |  | -0.106 | 0.330 |  | -0.047 | 0.670 |  | 0.171 | 0.115 |
| ApoB100 (g/L) | 0.404 | 0.000* |  | 0.223 | 0.039* |  | -0.097 | 0.374 |  | 0.253 | 0.019* |

Note: * indicates a P value less than 0.05. Abbreviation: C-reactive protein (CRP), syndecan-1 (SDC-1), platelets (PLT), interleukin 6 (IL-6), white blood cells (WBC), fibrinogen (FIB), activated partial thromboplastin time (APTT), thrombin time (TT), total cholesterol (TC), triglycerides (TG), high-density lipoprotein cholesterol (HDL-C), low density lipoprotein cholesterol (LDL-C), apolipoprotein A1 (ApoA1) and apolipoprotein B100 (ApoB100).

Table 4. SDC-1 levels in patients with different grades of CALs

|  | Mild CALs group (n=19) | Moderate CALs group (n=15) | Severe CALs group (n=7) | F | P |
| --- | --- | --- | --- | --- | --- |
| Coronary artery diameter (mm) | 4.19±2.80 | 6.57±3.18 | 9.30±2.55 | 8.468 | 0.001 |
| SDC-1 (ng/mL) | 5.52±0.87* | 6.18±0.54*# | 6.91±0.19# | 5.464 | 0.008 |

Note: * and # indicate a significant difference in the comparison. Abbreviation: syndecan-1 (SDC-1), coronary artery lesions (CALs).

Figure 3. ROC curve of SDC-1 diagnosis in CALs patients among KD patients. Abbreviation: Receiver operating characteristic (ROC), syndecan-1 (SDC-1), coronary artery lesions (CALs), Kawasaki disease (KD).

2. Correlation analysis:
   *  The correlation analysis is limited to a few selected parameters, such as PLT vs. LDL-C, IL-6 vs. TG, SDC-1 vs. TG, and CRP vs. ApoA1.
   *  Expanding the correlation analysis to include other potentially relevant variables (e.g., age, disease severity, treatment response) could provide a more comprehensive understanding of the relationships between the measured parameters

Reply: Thank you for the valuable suggestion, which is highly appreciated. In response to your request, we have expanded the sample size of this study, augmented the parameter analysis, and introduced additional tables. Furthermore, we have conducted a comprehensive reanalysis of the data throughout the manuscript.

**Changs in the text:**

**Methods**: 86 pediatric Kawasaki disease (KD) patients and 52 healthy controls admitted from January 2018 to December 2023 were retrospectively analyzed. Venous blood samples from KD patients were analyzed for white blood cells (WBC), platelets (PLT), C-reactive protein (CRP), interleukin-6 (IL-6), syndecan-1 (SDC-1), coagulation parameters, and lipid profiles. Correlations between these laboratory indicators were assessed. Receiver operating characteristic (ROC) curve analysis determined the diagnostic value of SDC-1 for coronary artery lesions (CALs) in KD patients. SDC-1 levels were further compared across different CAL severity groups.

**Results**: The levels of ALT, AST, WBC, PLT, CRP, IL-6, and SDC-1 in the KD group were significantly higher than those in the control group (P<0.05). Coagulation function analysis showed that APTT and TT were significantly increased, while FIB levels were significantly decreased in the KD group compared with the control group (P<0.05). Lipid profile analysis revealed that TC, HDL-C, and ApoA1 were significantly decreased, whereas TG, LDL-C, and ApoB100 were significantly increased in the KD group (P<0.05). Refractory KD patients exhibited significantly higher levels of ALT, AST, CRP, WBC, and TG compared to responsive KD patients (P<0.05). Correlation analysis indicated a strong positive correlation between PLT and LDL-C (r=0.813, P<0.001) and between IL-6 and TG (r=0.926, P<0.001), while CRP was negatively correlated with ApoA1 (r=-0.599, P=0.000). Among the 86 KD patients, 41 (47.67%) developed CALs, with 19 classified as mild, 15 as moderate, and 7 as severe. For predicting CALs among KD patients, the threshold of SDC-1 was identified as 5.5 ng/ml, with a sensitivity of 70.7%, specificity of 64.4%, positive predictive value of 65.91%, negative predictive value of 69.05%, and an AUC of 0.762 (95% confidence interval 0.662-0.861, P<0.001). SDC-1 levels significantly differed among the CAL severity groups (P<0.05), with higher levels observed in moderate compared to mild CALs, and in severe compared to moderate CALs (P<0.05). (page 2-3, line 35-61)

There were 86 subjects in the KD group, including 48 males (55.81%) and 38 females (44.19%). The age range was 3 months to 8.5 years, with a mean age of 3.34±1.59 years. There were 52 subjects in the control group, including 25 males (48.08%) and 27 females (51.92%). The age range was 4 months to 8.7 years, with a mean age of 3.48±1.60 years. (page 6-7, line 180-184)

Additionally, in liver function parameters, AST and ALT levels in the KD group were significantly higher than those in the control group (P<0.05). (Table 1). It is noteworthy that, compared with the refractory KD group, the levels of ALT, AST, CRP, WBC count and TG were significantly reduced in the responsive KD group (P<0.05). (Table 2) (page 7, line 191-195)

In the correlation analysis, we primarily examined the relationships between general laboratory indicators and lipid profiles. The results demonstrated a strong positive correlation between PLT and LDL-C (r=0.813, P<0.001) as well as IL-6 and TG (r=0.926, P<0.001). A moderate positive correlation was observed between SDC-1 and TG (r=0.485, P=0.034) and between IL-6 and ApoB100 (r=0.487, P=0.033). Additionally, there was a moderate negative correlation between CRP and ApoA1 (r=-0.487, P=0.033), with a stronger negative correlation observed between CRP and ApoA1 (r=-0.599, P=0.000). Moreover, a weaker correlation was found between IL-6 and TG (r=0.229, P=0.034) (Table 3) (page 7, line 198-206)

Among 86 KD patients in this study, 41 (47.67%) developed CALs. For predicting CALs among KD patients, the threshold of SDC-1 was identified as 5.5 ng/ml, with a sensitivity of 70.7%, specificity of 64.4%, positive predictive value of 65.91%, negative predictive value of 69.05%, and an AUC of 0.762 (95% confidence interval 0.662-0.861, P<0.001). The ROC curve of SDC-1 diagnosis in CALs patients among KD patients was presented in Figure 3. (page 7-8, line 208-213)

Of the 41 patients with CALs, 19 (46.34%) had mild CALs, 15 (36.59%) had moderate CALs, and 7 (17.07%) had severe CALs. Their mean coronary artery diameters were 4.19±2.80 mm, 6.57±3.18 mm, and 9.30±2.55 mm (page 8, line 215-217)

Table 1. Comparison of baseline clinical characteristics between KD group and control group

| Clinical characteristics | KD group (n=86) | Control group (n=52) | χ2/t | P |
| --- | --- | --- | --- | --- |
| Male (%) | 48（55.82%） | 25（48.08%） | 0.779 | 0.378 |
| Age (years) | 3.34±1.59 | 3.48±1.60 | -0.489 | 0.625 |
| Weight (kg) | 15.30±3.11 | 15.23±3.07 | 0.125 | 0.901 |
| ALT(U/L) | 77.14±23.46 | 26.35±10.37 | 14.748 | 0.000 |
| AST(U/L) | 65.71±17.80 | 28.81±8.73 | 13.957 | 0.000 |
| SDC-1 (ng/mL) | 5.52±1.02 | 2.51±0.65 | 19.030 | <0.001* |
| CRP (mg/L) | 62.53±23.88 | 5.69±1.60 | 18.012 | <0.001* |
| IL-6 (pg/mL) | 71.54±10.90 | 5.02±2.10 | 43.479 | <0.001* |
| WBC (×109/L) | 15.76±2.28 | 6.57±0.88 | 27.830 | <0.001* |
| PLT (×109/L) | 354.84±58.75 | 244.31±38.90 | 12.054 | <0.001* |
| APTT (s) | 33.02±5.75 | 21.23±1.64 | 14.430 | <0.001* |
| TT (s) | 16.14±2.99 | 14.40±2.65 | 3.442 | 0.001 |
| FIB (g/L) | 2.53±1.01 | 4.91±1.43 | -11.455 | <0.001* |
| TC (mmol/L) | 3.36±0.61 | 4.23±0.65 | -7.849 | <0.001* |
| TG (mmol/L) | 1.62±0.54 | 0.87±0.25 | 9.419 | <0.001* |
| HDL-C (mmol/L) | 0.84±0.18 | 1.50±0.31 | -15.789 | <0.001* |
| LDL-C (mmol/L) | 2.25±0.45 | 1.97±0.59 | 3.159 | 0.002 |
| ApoA1 (g/L) | 0.87±0.25 | 1.36±0.28 | -10.614 | 0.000 |
| ApoB100 (g/L) | 0.98±0.33 | 0.77±0.18 | 4.104 | <0.001* |

Note: * indicates a P value less than 0.05. Abbreviation: C-reactive protein (CRP), syndecan-1 (SDC-1), white blood cells (WBC), platelets (PLT), interleukin 6 (IL-6), thrombin time (TT), activated partial thromboplastin time (APTT), fibrinogen (FIB), total cholesterol (TC), triglycerides (TG), high-density lipoprotein cholesterol (HDL-C), low density lipoprotein cholesterol (LDL-C), apolipoprotein A1 (ApoA1) and apolipoprotein B100 (ApoB100).

Table 2. Comparison of baseline clinical characteristics between refractory KD group and responsive KD group

| Clinical characteristics | Refractory KD (n= 31) | Responsive KD  (n=55) | χ2/t | P |
| --- | --- | --- | --- | --- |
| Male (%) | 20(51.61%) | 28(43.64%) | 1.488 | 0.223 |
| Age (years) | 3.36±1.65 | 3.33±1.57 | 0.105 | 0.917 |
| Weight (kg) | 15.44±3.17 | 15.22±3.11 | 0.871 | 0.756 |
| ALT(U/L) | 86.45±23.19 | 71.89±22.13 | -2.879 | 0.005 |
| AST(U/L) | 72.71±16.65 | 61.76±17.34 | -2.851 | 0.005 |
| SDC-1 (ng/mL) | 6.24±0.77 | 5.12±0.93 | 5.690 | 0.000 |
| CRP (mg/L) | 72.80±34.52 | 61.44±13.74 | 2.163 | 0.033 |
| IL-6 (pg/mL) | 74.12±13.53 | 70.09±8.90 | 1.665 | 0.100 |
| WBC (×109/L) | 16.48±2.29 | 15.35±2.19 | 2.278 | 0.025 |
| PLT (×109/L) | 363.48±56.85 | 349.96±59.75 | 1.025 | 0.308 |
| APTT (s) | 34.03±6.34 | 32.45±5.36 | 1.226 | 0.224 |
| TT (s) | 16.03±3.04 | 16.20±3.00 | -0.248 | 0.805 |
| FIB (g/L) | 2.65±1.52 | 2.46±0.54 | 0.859 | 0.393 |
| TC (mmol/L) | 3.31±0.66 | 3.39±0.59 | -0.639 | 0.525 |
| TG (mmol/L) | 1.86±0.61 | 1.48±0.44 | 3.316 | 0.001 |
| HDL-C (mmol/L) | 0.83±0.19 | 0.84±0.18 | -0.264 | 0.792 |
| LDL-C (mmol/L) | 2.27±0.42 | 2.23±0.47 | 0.377 | 0.707 |
| ApoA1 (g/L) | 0.87±0.25 | 0.88±0.25 | -0.185 | 0.853 |
| ApoB100 (g/L) | 1.05±0.39 | 0.93±0.29 | 1.643 | 0.104 |

Note: * indicates a P value less than 0.05. Abbreviation: C-reactive protein (CRP), syndecan-1 (SDC-1), white blood cells (WBC), platelets (PLT), interleukin 6 (IL-6), thrombin time (TT), activated partial thromboplastin time (APTT), fibrinogen (FIB), total cholesterol (TC), triglycerides (TG), high-density lipoprotein cholesterol (HDL-C), low density lipoprotein cholesterol (LDL-C), apolipoprotein A1 (ApoA1) and apolipoprotein B100 (ApoB100).

Table 3. Correlation analysis of general laboratory indicators and lipid indicators

|  | CRP (mg/L) | |  | SDC-1 | |  | PLT (×109/L) | |  | IL-6 (pg/mL) | |
| --- | --- | --- | --- | --- | --- | --- | --- | --- | --- | --- | --- |
|  | r | P |  | r | P |  | r | P |  | r | P |
| WBC(×109/L) | 0.135 | 0.217 |  | 0.035 | 0.749 |  | 0.085 | 0.438 |  | -0.032 | 0.773 |
| FIB(g/L) | 0.475 | 0.000* |  | 0.008 | 0.941 |  | 0.104 | 0.342 |  | 0.342 | 0.001* |
| APTT (s) | 0.150 | 0.168 |  | -0.039 | 0.722 |  | 0.018 | 0.873 |  | 0.216 | 0.045* |
| TT (s) | -0.097 | 0.372 |  | 0.035 | 0.751 |  | 0.080 | 0.466 |  | 0.009 | 0.932 |
| TC (mmol/L) | 0.001 | 0.990 |  | -0.047 | 0.664 |  | -0.008 | 0.944 |  | -0.155 | 0.155 |
| TG (mmol/L) | 0.223 | 0.039* |  | 0.250 | 0.020* |  | 0.002 | 0.987 |  | 0.491 | 0.000* |
| HDL-C (mmol/L) | 0.048 | 0.659 |  | -0.149 | 0.171 |  | 0.025 | 0.817 |  | 0.022 | 0.839 |
| LDL-C (mmol/L) | -0.243 | 0.024* |  | 0.000 | 0.993 |  | 0.227 | 0.035 |  | 0.004 | 0.972 |
| ApoA1 (g/L) | -0.265 | 0.014* |  | -0.106 | 0.330 |  | -0.047 | 0.670 |  | 0.171 | 0.115 |
| ApoB100 (g/L) | 0.404 | 0.000* |  | 0.223 | 0.039* |  | -0.097 | 0.374 |  | 0.253 | 0.019* |

Note: * indicates a P value less than 0.05. Abbreviation: C-reactive protein (CRP), syndecan-1 (SDC-1), platelets (PLT), interleukin 6 (IL-6), white blood cells (WBC), fibrinogen (FIB), activated partial thromboplastin time (APTT), thrombin time (TT), total cholesterol (TC), triglycerides (TG), high-density lipoprotein cholesterol (HDL-C), low density lipoprotein cholesterol (LDL-C), apolipoprotein A1 (ApoA1) and apolipoprotein B100 (ApoB100).

Table 4. SDC-1 levels in patients with different grades of CALs

|  | Mild CALs group (n=19) | Moderate CALs group (n=15) | Severe CALs group (n=7) | F | P |
| --- | --- | --- | --- | --- | --- |
| Coronary artery diameter (mm) | 4.19±2.80 | 6.57±3.18 | 9.30±2.55 | 8.468 | 0.001 |
| SDC-1 (ng/mL) | 5.52±0.87* | 6.18±0.54*# | 6.91±0.19# | 5.464 | 0.008 |

Note: * and # indicate a significant difference in the comparison. Abbreviation: syndecan-1 (SDC-1), coronary artery lesions (CALs).

Figure 3. ROC curve of SDC-1 diagnosis in CALs patients among KD patients. Abbreviation: Receiver operating characteristic (ROC), syndecan-1 (SDC-1), coronary artery lesions (CALs), Kawasaki disease (KD).

3.  Subgroup analysis:
*  The section on "SDC-1 levels in patients with different grades of CALs" provides valuable insights into the association between SDC-1 and the severity of coronary artery lesions.
*  However, the sample size for the subgroups (mild, moderate, and severe CALs) is quite small, which may limit the reliability of the comparisons.
*  Considering a larger sample size or performing a sensitivity analysis to assess the robustness of these subgroup findings would strengthen the conclusions.

Reply: Thank you for your comments and valuable feedback on the section "SDC-1 levels in patients with different grades of CALs" in our study. Your observation regarding the potential limitations in the reliability of comparisons due to the smaller subgroup sizes is indeed well-founded.

We acknowledge that the subgroups of patients with varying grades of CALs (mild, moderate, and severe) in our study had a limited sample size, which could indeed affect the statistical power and reliability of our conclusions.

Therefore, we have added the sample size as suggested. Nevertheless, we employed rigorous statistical methods in our analysis, including the Kolmogorov-Smirnov test for normality and appropriate analysis of variance, to ensure the scientific validity and reliability of our results.

Despite the limited sample size, our results provide preliminary evidence of a significant association between SDC-1 levels and the severity of CALs, which could have important implications for the clinical management of patients with KD.

Taking into account your suggestions, we have supplemented the sample size in the revised manuscript and may conduct sensitivity analyses to assess the robustness of our findings and further validate the potential of SDC-1 as a biomarker for the severity of CALs. The relevant content has been mentioned in the limitation.

We welcome any further recommendations you may have on improving our research design and analytical methods. We believe that through collaboration with you and other colleagues, we can enhance the quality of our research and provide stronger scientific evidence for the diagnosis and treatment of KD patients.Once again, thank you for your valuable feedback and ongoing support.

**Changs in the text:**

**Methods**: 86 pediatric Kawasaki disease (KD) patients and 52 healthy controls admitted from January 2018 to December 2023 were retrospectively analyzed. Venous blood samples from KD patients were analyzed for white blood cells (WBC), platelets (PLT), C-reactive protein (CRP), interleukin-6 (IL-6), syndecan-1 (SDC-1), coagulation parameters, and lipid profiles. Correlations between these laboratory indicators were assessed. Receiver operating characteristic (ROC) curve analysis determined the diagnostic value of SDC-1 for coronary artery lesions (CALs) in KD patients. SDC-1 levels were further compared across different CAL severity groups.

**Results**: The levels of ALT, AST, WBC, PLT, CRP, IL-6, and SDC-1 in the KD group were significantly higher than those in the control group (P<0.05). Coagulation function analysis showed that APTT and TT were significantly increased, while FIB levels were significantly decreased in the KD group compared with the control group (P<0.05). Lipid profile analysis revealed that TC, HDL-C, and ApoA1 were significantly decreased, whereas TG, LDL-C, and ApoB100 were significantly increased in the KD group (P<0.05). Refractory KD patients exhibited significantly higher levels of ALT, AST, CRP, WBC, and TG compared to responsive KD patients (P<0.05). Correlation analysis indicated a strong positive correlation between PLT and LDL-C (r=0.813, P<0.001) and between IL-6 and TG (r=0.926, P<0.001), while CRP was negatively correlated with ApoA1 (r=-0.599, P=0.000). Among the 86 KD patients, 41 (47.67%) developed CALs, with 19 classified as mild, 15 as moderate, and 7 as severe. For predicting CALs among KD patients, the threshold of SDC-1 was identified as 5.5 ng/ml, with a sensitivity of 70.7%, specificity of 64.4%, positive predictive value of 65.91%, negative predictive value of 69.05%, and an AUC of 0.762 (95% confidence interval 0.662-0.861, P<0.001). SDC-1 levels significantly differed among the CAL severity groups (P<0.05), with higher levels observed in moderate compared to mild CALs, and in severe compared to moderate CALs (P<0.05). (page 2-3, line 35-61)

There were 86 subjects in the KD group, including 48 males (55.81%) and 38 females (44.19%). The age range was 3 months to 8.5 years, with a mean age of 3.34±1.59 years. There were 52 subjects in the control group, including 25 males (48.08%) and 27 females (51.92%). The age range was 4 months to 8.7 years, with a mean age of 3.48±1.60 years. (page 6-7, line 180-184)

Additionally, in liver function parameters, AST and ALT levels in the KD group were significantly higher than those in the control group (P<0.05). (Table 1). It is noteworthy that, compared with the refractory KD group, the levels of ALT, AST, CRP, WBC count and TG were significantly reduced in the responsive KD group (P<0.05). (Table 2) (page 7, line 191-195)

In the correlation analysis, we primarily examined the relationships between general laboratory indicators and lipid profiles. The results demonstrated a strong positive correlation between PLT and LDL-C (r=0.813, P<0.001) as well as IL-6 and TG (r=0.926, P<0.001). A moderate positive correlation was observed between SDC-1 and TG (r=0.485, P=0.034) and between IL-6 and ApoB100 (r=0.487, P=0.033). Additionally, there was a moderate negative correlation between CRP and ApoA1 (r=-0.487, P=0.033), with a stronger negative correlation observed between CRP and ApoA1 (r=-0.599, P=0.000). Moreover, a weaker correlation was found between IL-6 and TG (r=0.229, P=0.034) (Table 3) (page 7, line 198-206)

Among 86 KD patients in this study, 41 (47.67%) developed CALs. For predicting CALs among KD patients, the threshold of SDC-1 was identified as 5.5 ng/ml, with a sensitivity of 70.7%, specificity of 64.4%, positive predictive value of 65.91%, negative predictive value of 69.05%, and an AUC of 0.762 (95% confidence interval 0.662-0.861, P<0.001). The ROC curve of SDC-1 diagnosis in CALs patients among KD patients was presented in Figure 3. (page 7-8, line 208-213)

Of the 41 patients with CALs, 19 (46.34%) had mild CALs, 15 (36.59%) had moderate CALs, and 7 (17.07%) had severe CALs. Their mean coronary artery diameters were 4.19±2.80 mm, 6.57±3.18 mm, and 9.30±2.55 mm (page 8, line 215-217)

Table 1. Comparison of baseline clinical characteristics between KD group and control group

| Clinical characteristics | KD group (n=86) | Control group (n=52) | χ2/t | P |
| --- | --- | --- | --- | --- |
| Male (%) | 48（55.82%） | 25（48.08%） | 0.779 | 0.378 |
| Age (years) | 3.34±1.59 | 3.48±1.60 | -0.489 | 0.625 |
| Weight (kg) | 15.30±3.11 | 15.23±3.07 | 0.125 | 0.901 |
| ALT(U/L) | 77.14±23.46 | 26.35±10.37 | 14.748 | 0.000 |
| AST(U/L) | 65.71±17.80 | 28.81±8.73 | 13.957 | 0.000 |
| SDC-1 (ng/mL) | 5.52±1.02 | 2.51±0.65 | 19.030 | <0.001* |
| CRP (mg/L) | 62.53±23.88 | 5.69±1.60 | 18.012 | <0.001* |
| IL-6 (pg/mL) | 71.54±10.90 | 5.02±2.10 | 43.479 | <0.001* |
| WBC (×109/L) | 15.76±2.28 | 6.57±0.88 | 27.830 | <0.001* |
| PLT (×109/L) | 354.84±58.75 | 244.31±38.90 | 12.054 | <0.001* |
| APTT (s) | 33.02±5.75 | 21.23±1.64 | 14.430 | <0.001* |
| TT (s) | 16.14±2.99 | 14.40±2.65 | 3.442 | 0.001 |
| FIB (g/L) | 2.53±1.01 | 4.91±1.43 | -11.455 | <0.001* |
| TC (mmol/L) | 3.36±0.61 | 4.23±0.65 | -7.849 | <0.001* |
| TG (mmol/L) | 1.62±0.54 | 0.87±0.25 | 9.419 | <0.001* |
| HDL-C (mmol/L) | 0.84±0.18 | 1.50±0.31 | -15.789 | <0.001* |
| LDL-C (mmol/L) | 2.25±0.45 | 1.97±0.59 | 3.159 | 0.002 |
| ApoA1 (g/L) | 0.87±0.25 | 1.36±0.28 | -10.614 | 0.000 |
| ApoB100 (g/L) | 0.98±0.33 | 0.77±0.18 | 4.104 | <0.001* |

Note: * indicates a P value less than 0.05. Abbreviation: C-reactive protein (CRP), syndecan-1 (SDC-1), white blood cells (WBC), platelets (PLT), interleukin 6 (IL-6), thrombin time (TT), activated partial thromboplastin time (APTT), fibrinogen (FIB), total cholesterol (TC), triglycerides (TG), high-density lipoprotein cholesterol (HDL-C), low density lipoprotein cholesterol (LDL-C), apolipoprotein A1 (ApoA1) and apolipoprotein B100 (ApoB100).

Table 2. Comparison of baseline clinical characteristics between refractory KD group and responsive KD group

| Clinical characteristics | Refractory KD (n= 31) | Responsive KD  (n=55) | χ2/t | P |
| --- | --- | --- | --- | --- |
| Male (%) | 20(51.61%) | 28(43.64%) | 1.488 | 0.223 |
| Age (years) | 3.36±1.65 | 3.33±1.57 | 0.105 | 0.917 |
| Weight (kg) | 15.44±3.17 | 15.22±3.11 | 0.871 | 0.756 |
| ALT(U/L) | 86.45±23.19 | 71.89±22.13 | -2.879 | 0.005 |
| AST(U/L) | 72.71±16.65 | 61.76±17.34 | -2.851 | 0.005 |
| SDC-1 (ng/mL) | 6.24±0.77 | 5.12±0.93 | 5.690 | 0.000 |
| CRP (mg/L) | 72.80±34.52 | 61.44±13.74 | 2.163 | 0.033 |
| IL-6 (pg/mL) | 74.12±13.53 | 70.09±8.90 | 1.665 | 0.100 |
| WBC (×109/L) | 16.48±2.29 | 15.35±2.19 | 2.278 | 0.025 |
| PLT (×109/L) | 363.48±56.85 | 349.96±59.75 | 1.025 | 0.308 |
| APTT (s) | 34.03±6.34 | 32.45±5.36 | 1.226 | 0.224 |
| TT (s) | 16.03±3.04 | 16.20±3.00 | -0.248 | 0.805 |
| FIB (g/L) | 2.65±1.52 | 2.46±0.54 | 0.859 | 0.393 |
| TC (mmol/L) | 3.31±0.66 | 3.39±0.59 | -0.639 | 0.525 |
| TG (mmol/L) | 1.86±0.61 | 1.48±0.44 | 3.316 | 0.001 |
| HDL-C (mmol/L) | 0.83±0.19 | 0.84±0.18 | -0.264 | 0.792 |
| LDL-C (mmol/L) | 2.27±0.42 | 2.23±0.47 | 0.377 | 0.707 |
| ApoA1 (g/L) | 0.87±0.25 | 0.88±0.25 | -0.185 | 0.853 |
| ApoB100 (g/L) | 1.05±0.39 | 0.93±0.29 | 1.643 | 0.104 |

Note: * indicates a P value less than 0.05. Abbreviation: C-reactive protein (CRP), syndecan-1 (SDC-1), white blood cells (WBC), platelets (PLT), interleukin 6 (IL-6), thrombin time (TT), activated partial thromboplastin time (APTT), fibrinogen (FIB), total cholesterol (TC), triglycerides (TG), high-density lipoprotein cholesterol (HDL-C), low density lipoprotein cholesterol (LDL-C), apolipoprotein A1 (ApoA1) and apolipoprotein B100 (ApoB100).

Table 3. Correlation analysis of general laboratory indicators and lipid indicators

|  | CRP (mg/L) | |  | SDC-1 | |  | PLT (×109/L) | |  | IL-6 (pg/mL) | |
| --- | --- | --- | --- | --- | --- | --- | --- | --- | --- | --- | --- |
|  | r | P |  | r | P |  | r | P |  | r | P |
| WBC(×109/L) | 0.135 | 0.217 |  | 0.035 | 0.749 |  | 0.085 | 0.438 |  | -0.032 | 0.773 |
| FIB(g/L) | 0.475 | 0.000* |  | 0.008 | 0.941 |  | 0.104 | 0.342 |  | 0.342 | 0.001* |
| APTT (s) | 0.150 | 0.168 |  | -0.039 | 0.722 |  | 0.018 | 0.873 |  | 0.216 | 0.045* |
| TT (s) | -0.097 | 0.372 |  | 0.035 | 0.751 |  | 0.080 | 0.466 |  | 0.009 | 0.932 |
| TC (mmol/L) | 0.001 | 0.990 |  | -0.047 | 0.664 |  | -0.008 | 0.944 |  | -0.155 | 0.155 |
| TG (mmol/L) | 0.223 | 0.039* |  | 0.250 | 0.020* |  | 0.002 | 0.987 |  | 0.491 | 0.000* |
| HDL-C (mmol/L) | 0.048 | 0.659 |  | -0.149 | 0.171 |  | 0.025 | 0.817 |  | 0.022 | 0.839 |
| LDL-C (mmol/L) | -0.243 | 0.024* |  | 0.000 | 0.993 |  | 0.227 | 0.035 |  | 0.004 | 0.972 |
| ApoA1 (g/L) | -0.265 | 0.014* |  | -0.106 | 0.330 |  | -0.047 | 0.670 |  | 0.171 | 0.115 |
| ApoB100 (g/L) | 0.404 | 0.000* |  | 0.223 | 0.039* |  | -0.097 | 0.374 |  | 0.253 | 0.019* |

Note: * indicates a P value less than 0.05. Abbreviation: C-reactive protein (CRP), syndecan-1 (SDC-1), platelets (PLT), interleukin 6 (IL-6), white blood cells (WBC), fibrinogen (FIB), activated partial thromboplastin time (APTT), thrombin time (TT), total cholesterol (TC), triglycerides (TG), high-density lipoprotein cholesterol (HDL-C), low density lipoprotein cholesterol (LDL-C), apolipoprotein A1 (ApoA1) and apolipoprotein B100 (ApoB100).

Table 4. SDC-1 levels in patients with different grades of CALs

|  | Mild CALs group (n=19) | Moderate CALs group (n=15) | Severe CALs group (n=7) | F | P |
| --- | --- | --- | --- | --- | --- |
| Coronary artery diameter (mm) | 4.19±2.80 | 6.57±3.18 | 9.30±2.55 | 8.468 | 0.001 |
| SDC-1 (ng/mL) | 5.52±0.87* | 6.18±0.54*# | 6.91±0.19# | 5.464 | 0.008 |

Note: * and # indicate a significant difference in the comparison. Abbreviation: syndecan-1 (SDC-1), coronary artery lesions (CALs).

Figure 3. ROC curve of SDC-1 diagnosis in CALs patients among KD patients. Abbreviation: Receiver operating characteristic (ROC), syndecan-1 (SDC-1), coronary artery lesions (CALs), Kawasaki disease (KD).

4.  Potential confounding factors:
*  The results section does not address the potential impact of confounding factors, such as disease duration, treatment status, or comorbidities, on the measured parameters.
*  Adjusting for relevant confounders in the analyses could provide a more accurate estimation of the associations observed.
Reply: First and foremost, we extend our sincere gratitude for the valuable feedback on our study. The suggestion to consider the potential impact of confounding factors such as disease duration, treatment status or comorbidities on the measured parameters is indeed insightful. In our study, we conducted statistical analyses on factors such as age, gender and weight, and found no statistically significant differences among the groups in our study. This suggests that the impact of these variables on the study outcomes within our sample may be limited. We have added the relevant content into the discussion as suggested.

However, in the current study, we acknowledge that the diversity and variability of treatment status made it challenging to control for this variable in our analysis. Additionally, due to limitations in funding and resources, we were unable to collect detailed data on disease duration, and thus, we could not adequately adjust for these potential confounders:

Despite these limitations, we recognize that these factors may exert a certain influence on the study outcomes. For instance, the duration of disease may affect the levels of inflammatory markers, while changes in treatment status could influence patients' laboratory indicators.

In light of your suggestions, we will consider designing prospective studies in the future to collect more comprehensive data, including regular updates on disease duration, and incorporate treatment response as a covariate in the analysis to assess its potential impact on the results.

We believe that these approaches will enable future studies to more accurately estimate the observed associations and provide stronger evidence for clinical practice. We have acknowledged these issues in the limitations section of our manuscript.

Once again, we appreciate your valuable comments and look forward to further communication and guidance.

**Changs in the text:**

In our study, we conducted statistical analyses on factors such as age, gender and weight, and found no statistically significant differences among the groups in our study. This suggests that the impact of these variables on the study outcomes within our sample may be limited. (page 11-12, line 330-333)

On the other hand, the diversity and variability of treatment status present challenges in controlling for this variable in the current analysis. What’s more, due to constraints on funding and resources, our study did not collect detailed data on disease duration. In future research, we will consider designing prospective studies to gather more comprehensive data, including regular updates on disease duration. We also plan to include treatment response as a covariate in the analysis to assess its potential impact on the outcomes. (page 12, line 340-346)

¶Discussion

Well written and organized, some weak points should be addressed.
*  Oversimplification of mechanisms: The explanations for the observed lipid and coagulation disturbances, while plausible, may oversimplify the complex underlying mechanisms. The text could benefit from a more nuanced discussion of the specific inflammatory pathways, signaling cascades, and regulatory factors involved in these metabolic and hemostatic alterations.

Reply: Thank you for taking time to review our article and provide the valuable suggstion, which is highly appreciated.

**Changs in the text:**

Syndecan-1 (SDC-1) is a transmembrane heparan sulfate proteoglycan predominantly found on the glycocalyx of epithelial and endothelial cells. Its ectodomain can be cleaved by matrix metalloproteinases and released into the serum, where elevated levels serve as biomarkers for endothelial activation and damage in various conditions.

SDC-1 is involved in various cellular processes, including cell proliferation, apoptosis, and inflammation. It plays a crucial role in modulating the vascular endothelial barrier function and inflammatory response, both of which are central to the pathophysiology of KD. SDC-1 interacts with several signaling molecules, including growth factors, cytokines, and chemokines, facilitating their binding to their respective receptors, thereby modulating downstream signaling pathways such as the MAPK/ERK^29^ and NF-κB pathways ^30^. These pathways are known to influence inflammatory processes and endothelial cell function, which are critical in the development of CALs.

In KD, the endothelial damage and increased vascular permeability contribute to the formation of coronary aneurysms. SDC-1 shedding, induced by pro-inflammatory cytokines, results in the release of its ectodomain, which can act as a soluble receptor, further exacerbating the inflammatory response and endothelial dysfunction. The loss of SDC-1 from the endothelial surface also leads to a breakdown of the glycocalyx, a protective layer on the endothelial cells, making the coronary arteries more susceptible to damage and aneurysm formation.

In our study, we have observed that elevated levels of SDC-1 are correlated with the severity of CALs in KD patients, suggesting that SDC-1 may serve as a potential biomarker for predicting the development and progression of CALs. The detailed mechanistic insights into SDC-1's role in KD provided in the revised manuscript aim to offer a more comprehensive understanding of its involvement in disease pathogenesis and its potential as a therapeutic target. (page 10-11, line 285-310)

**References**

29. Sun H, Hu Y, Gu Z, Owens RT, Chen YQ, Edwards IJ. Omega-3 fatty acids induce apoptosis in human breast cancer cells and mouse mammary tissue through syndecan-1 inhibition of the MEK-Erk pathway. Carcinogenesis. 2011 Oct;32(10):1518-24.

30. Lin L, Qiu D, Yang F, Xia Y, Cai S, Liao X, Deng W, Wu C. Hydrogen-rich saline upregulates the Sirt1/NF-κB signaling pathway and reduces vascular endothelial glycocalyx shedding in sepsis-induced acute kidney injury. Shock. 2024 May 27. (page 17, line 490-497)

*  In lines 48 to 50 in the discussion, the author addressed liver functions impairments. So I wonder why liver functions panel weren't involved as biomarkers in this study?

Reply: Thank you for the valuable suggestion, which help improve the accuracy of the manuscript. The relevant content has been added into the text as suggested.

**Changs in the text:**

**Methods**: 86 pediatric Kawasaki disease (KD) patients and 52 healthy controls admitted from January 2018 to December 2023 were retrospectively analyzed. Venous blood samples from KD patients were analyzed for white blood cells (WBC), platelets (PLT), C-reactive protein (CRP), interleukin-6 (IL-6), syndecan-1 (SDC-1), coagulation parameters, and lipid profiles. Correlations between these laboratory indicators were assessed. Receiver operating characteristic (ROC) curve analysis determined the diagnostic value of SDC-1 for coronary artery lesions (CALs) in KD patients. SDC-1 levels were further compared across different CAL severity groups.

**Results**: The levels of ALT, AST, WBC, PLT, CRP, IL-6, and SDC-1 in the KD group were significantly higher than those in the control group (P<0.05). Coagulation function analysis showed that APTT and TT were significantly increased, while FIB levels were significantly decreased in the KD group compared with the control group (P<0.05). Lipid profile analysis revealed that TC, HDL-C, and ApoA1 were significantly decreased, whereas TG, LDL-C, and ApoB100 were significantly increased in the KD group (P<0.05). Refractory KD patients exhibited significantly higher levels of ALT, AST, CRP, WBC, and TG compared to responsive KD patients (P<0.05). Correlation analysis indicated a strong positive correlation between PLT and LDL-C (r=0.813, P<0.001) and between IL-6 and TG (r=0.926, P<0.001), while CRP was negatively correlated with ApoA1 (r=-0.599, P=0.000). Among the 86 KD patients, 41 (47.67%) developed CALs, with 19 classified as mild, 15 as moderate, and 7 as severe. For predicting CALs among KD patients, the threshold of SDC-1 was identified as 5.5 ng/ml, with a sensitivity of 70.7%, specificity of 64.4%, positive predictive value of 65.91%, negative predictive value of 69.05%, and an AUC of 0.762 (95% confidence interval 0.662-0.861, P<0.001). SDC-1 levels significantly differed among the CAL severity groups (P<0.05), with higher levels observed in moderate compared to mild CALs, and in severe compared to moderate CALs (P<0.05). (page 2-3, line 35-61)

There were 86 subjects in the KD group, including 48 males (55.81%) and 38 females (44.19%). The age range was 3 months to 8.5 years, with a mean age of 3.34±1.59 years. There were 52 subjects in the control group, including 25 males (48.08%) and 27 females (51.92%). The age range was 4 months to 8.7 years, with a mean age of 3.48±1.60 years. (page 6-7, line 180-184)

Additionally, in liver function parameters, AST and ALT levels in the KD group were significantly higher than those in the control group (P<0.05). (Table 1). It is noteworthy that, compared with the refractory KD group, the levels of ALT, AST, CRP, WBC count and TG were significantly reduced in the responsive KD group (P<0.05). (Table 2) (page 7, line 191-195)

In the correlation analysis, we primarily examined the relationships between general laboratory indicators and lipid profiles. The results demonstrated a strong positive correlation between PLT and LDL-C (r=0.813, P<0.001) as well as IL-6 and TG (r=0.926, P<0.001). A moderate positive correlation was observed between SDC-1 and TG (r=0.485, P=0.034) and between IL-6 and ApoB100 (r=0.487, P=0.033). Additionally, there was a moderate negative correlation between CRP and ApoA1 (r=-0.487, P=0.033), with a stronger negative correlation observed between CRP and ApoA1 (r=-0.599, P=0.000). Moreover, a weaker correlation was found between IL-6 and TG (r=0.229, P=0.034) (Table 3) (page 7, line 198-206)

Among 86 KD patients in this study, 41 (47.67%) developed CALs. For predicting CALs among KD patients, the threshold of SDC-1 was identified as 5.5 ng/ml, with a sensitivity of 70.7%, specificity of 64.4%, positive predictive value of 65.91%, negative predictive value of 69.05%, and an AUC of 0.762 (95% confidence interval 0.662-0.861, P<0.001). The ROC curve of SDC-1 diagnosis in CALs patients among KD patients was presented in Figure 3. (page 7-8, line 208-213)

Of the 41 patients with CALs, 19 (46.34%) had mild CALs, 15 (36.59%) had moderate CALs, and 7 (17.07%) had severe CALs. Their mean coronary artery diameters were 4.19±2.80 mm, 6.57±3.18 mm, and 9.30±2.55 mm (page 8, line 215-217)

Table 1. Comparison of baseline clinical characteristics between KD group and control group

| Clinical characteristics | KD group (n=86) | Control group (n=52) | χ2/t | P |
| --- | --- | --- | --- | --- |
| Male (%) | 48（55.82%） | 25（48.08%） | 0.779 | 0.378 |
| Age (years) | 3.34±1.59 | 3.48±1.60 | -0.489 | 0.625 |
| Weight (kg) | 15.30±3.11 | 15.23±3.07 | 0.125 | 0.901 |
| ALT(U/L) | 77.14±23.46 | 26.35±10.37 | 14.748 | 0.000 |
| AST(U/L) | 65.71±17.80 | 28.81±8.73 | 13.957 | 0.000 |
| SDC-1 (ng/mL) | 5.52±1.02 | 2.51±0.65 | 19.030 | <0.001* |
| CRP (mg/L) | 62.53±23.88 | 5.69±1.60 | 18.012 | <0.001* |
| IL-6 (pg/mL) | 71.54±10.90 | 5.02±2.10 | 43.479 | <0.001* |
| WBC (×109/L) | 15.76±2.28 | 6.57±0.88 | 27.830 | <0.001* |
| PLT (×109/L) | 354.84±58.75 | 244.31±38.90 | 12.054 | <0.001* |
| APTT (s) | 33.02±5.75 | 21.23±1.64 | 14.430 | <0.001* |
| TT (s) | 16.14±2.99 | 14.40±2.65 | 3.442 | 0.001 |
| FIB (g/L) | 2.53±1.01 | 4.91±1.43 | -11.455 | <0.001* |
| TC (mmol/L) | 3.36±0.61 | 4.23±0.65 | -7.849 | <0.001* |
| TG (mmol/L) | 1.62±0.54 | 0.87±0.25 | 9.419 | <0.001* |
| HDL-C (mmol/L) | 0.84±0.18 | 1.50±0.31 | -15.789 | <0.001* |
| LDL-C (mmol/L) | 2.25±0.45 | 1.97±0.59 | 3.159 | 0.002 |
| ApoA1 (g/L) | 0.87±0.25 | 1.36±0.28 | -10.614 | 0.000 |
| ApoB100 (g/L) | 0.98±0.33 | 0.77±0.18 | 4.104 | <0.001* |

Note: * indicates a P value less than 0.05. Abbreviation: C-reactive protein (CRP), syndecan-1 (SDC-1), white blood cells (WBC), platelets (PLT), interleukin 6 (IL-6), thrombin time (TT), activated partial thromboplastin time (APTT), fibrinogen (FIB), total cholesterol (TC), triglycerides (TG), high-density lipoprotein cholesterol (HDL-C), low density lipoprotein cholesterol (LDL-C), apolipoprotein A1 (ApoA1) and apolipoprotein B100 (ApoB100).

Table 2. Comparison of baseline clinical characteristics between refractory KD group and responsive KD group

| Clinical characteristics | Refractory KD (n= 31) | Responsive KD  (n=55) | χ2/t | P |
| --- | --- | --- | --- | --- |
| Male (%) | 20(51.61%) | 28(43.64%) | 1.488 | 0.223 |
| Age (years) | 3.36±1.65 | 3.33±1.57 | 0.105 | 0.917 |
| Weight (kg) | 15.44±3.17 | 15.22±3.11 | 0.871 | 0.756 |
| ALT(U/L) | 86.45±23.19 | 71.89±22.13 | -2.879 | 0.005 |
| AST(U/L) | 72.71±16.65 | 61.76±17.34 | -2.851 | 0.005 |
| SDC-1 (ng/mL) | 6.24±0.77 | 5.12±0.93 | 5.690 | 0.000 |
| CRP (mg/L) | 72.80±34.52 | 61.44±13.74 | 2.163 | 0.033 |
| IL-6 (pg/mL) | 74.12±13.53 | 70.09±8.90 | 1.665 | 0.100 |
| WBC (×109/L) | 16.48±2.29 | 15.35±2.19 | 2.278 | 0.025 |
| PLT (×109/L) | 363.48±56.85 | 349.96±59.75 | 1.025 | 0.308 |
| APTT (s) | 34.03±6.34 | 32.45±5.36 | 1.226 | 0.224 |
| TT (s) | 16.03±3.04 | 16.20±3.00 | -0.248 | 0.805 |
| FIB (g/L) | 2.65±1.52 | 2.46±0.54 | 0.859 | 0.393 |
| TC (mmol/L) | 3.31±0.66 | 3.39±0.59 | -0.639 | 0.525 |
| TG (mmol/L) | 1.86±0.61 | 1.48±0.44 | 3.316 | 0.001 |
| HDL-C (mmol/L) | 0.83±0.19 | 0.84±0.18 | -0.264 | 0.792 |
| LDL-C (mmol/L) | 2.27±0.42 | 2.23±0.47 | 0.377 | 0.707 |
| ApoA1 (g/L) | 0.87±0.25 | 0.88±0.25 | -0.185 | 0.853 |
| ApoB100 (g/L) | 1.05±0.39 | 0.93±0.29 | 1.643 | 0.104 |

Note: * indicates a P value less than 0.05. Abbreviation: C-reactive protein (CRP), syndecan-1 (SDC-1), white blood cells (WBC), platelets (PLT), interleukin 6 (IL-6), thrombin time (TT), activated partial thromboplastin time (APTT), fibrinogen (FIB), total cholesterol (TC), triglycerides (TG), high-density lipoprotein cholesterol (HDL-C), low density lipoprotein cholesterol (LDL-C), apolipoprotein A1 (ApoA1) and apolipoprotein B100 (ApoB100).

Table 3. Correlation analysis of general laboratory indicators and lipid indicators

|  | CRP (mg/L) | |  | SDC-1 | |  | PLT (×109/L) | |  | IL-6 (pg/mL) | |
| --- | --- | --- | --- | --- | --- | --- | --- | --- | --- | --- | --- |
|  | r | P |  | r | P |  | r | P |  | r | P |
| WBC(×109/L) | 0.135 | 0.217 |  | 0.035 | 0.749 |  | 0.085 | 0.438 |  | -0.032 | 0.773 |
| FIB(g/L) | 0.475 | 0.000* |  | 0.008 | 0.941 |  | 0.104 | 0.342 |  | 0.342 | 0.001* |
| APTT (s) | 0.150 | 0.168 |  | -0.039 | 0.722 |  | 0.018 | 0.873 |  | 0.216 | 0.045* |
| TT (s) | -0.097 | 0.372 |  | 0.035 | 0.751 |  | 0.080 | 0.466 |  | 0.009 | 0.932 |
| TC (mmol/L) | 0.001 | 0.990 |  | -0.047 | 0.664 |  | -0.008 | 0.944 |  | -0.155 | 0.155 |
| TG (mmol/L) | 0.223 | 0.039* |  | 0.250 | 0.020* |  | 0.002 | 0.987 |  | 0.491 | 0.000* |
| HDL-C (mmol/L) | 0.048 | 0.659 |  | -0.149 | 0.171 |  | 0.025 | 0.817 |  | 0.022 | 0.839 |
| LDL-C (mmol/L) | -0.243 | 0.024* |  | 0.000 | 0.993 |  | 0.227 | 0.035 |  | 0.004 | 0.972 |
| ApoA1 (g/L) | -0.265 | 0.014* |  | -0.106 | 0.330 |  | -0.047 | 0.670 |  | 0.171 | 0.115 |
| ApoB100 (g/L) | 0.404 | 0.000* |  | 0.223 | 0.039* |  | -0.097 | 0.374 |  | 0.253 | 0.019* |

Note: * indicates a P value less than 0.05. Abbreviation: C-reactive protein (CRP), syndecan-1 (SDC-1), platelets (PLT), interleukin 6 (IL-6), white blood cells (WBC), fibrinogen (FIB), activated partial thromboplastin time (APTT), thrombin time (TT), total cholesterol (TC), triglycerides (TG), high-density lipoprotein cholesterol (HDL-C), low density lipoprotein cholesterol (LDL-C), apolipoprotein A1 (ApoA1) and apolipoprotein B100 (ApoB100).

*  Also in same lines author should mention that there other references that suggest that systemic inflammation couldn't fully explain hepatic impairments(please read that https://onlinelibrary.wiley.com/doi/10.1002/ped4.12112)
Reply: Thank you for the valuable suggestion and the great reference. We strongly agree with your opinion and the relevant content and refernece have been added into the revised manuscript as suggested.

**Changs in the text:**

On the other hand, other references showed that systemic inflammation couldn't fully explain hepatic impairments.^28^ (page 10, line 274-275)

**References**

28.Tomita Y, Fukaya T, Yamaura Y, Tsujiguchi R, Muratani H, Shimaya M. Implications of hepatic dysfunction in Kawasaki disease: Time-related changes in aspartate aminotransferase, alanine aminotransferase, total bilirubin, and C-reactive protein levels. Pediatr Investig. 2019 Mar 22;3(1):19-26. (page 17, line 486-489)

*  I invite authors to read about relations of liver function panels in IVIG resistant KD and in CAD in KD, adding this point would be very valuable.

Reply: Thank you for the valuable suggestion. We strongly agree with your opinion and the relevant content have been added into the revised manuscript as follows:

**Changs in the text:**

Our study identified significantly elevated levels of AST and ALT in patients with refractory KD compared to the responsive KD. The liver's central role in immune modulation and systemic metabolism makes it particularly susceptible to inflammatory insults, which may exacerbate the overall disease severity in Kawasaki disease. From a mechanistic standpoint, the observed hepatic dysfunction in the refractory KD group likely reflects a heightened inflammatory burden. Kawasaki disease pathogenesis involves systemic vasculitis and endothelial activation, processes that can significantly impact hepatic function due to the liver's role in detoxification and cytokine metabolism. The elevated hepatic enzymes observed may be indicative of an exacerbated inflammatory response, with the liver potentially acting as both a target and a mediator of systemic inflammation. This is further supported by the increased levels of pro-inflammatory cytokines, such as CRP and IL-6, which have been implicated in both liver injury and the pathophysiology of Kawasaki disease. (page 9, line 243-256)

*  Absence of clinical implications: The discussion focuses on the pathophysiological changes in KD but does not explicitly address the clinical significance or management implications of these findings. Discussing the potential impact on patient outcomes, treatment strategies, and areas for further research could strengthen the practical relevance of the discussion.

Reply: Thank you for the valuable suggestion. We have revised it as suggested.

**Changs in the text:**

Syndecan-1 (SDC-1) is a transmembrane heparan sulfate proteoglycan predominantly found on the glycocalyx of epithelial and endothelial cells. Its ectodomain can be cleaved by matrix metalloproteinases and released into the serum, where elevated levels serve as biomarkers for endothelial activation and damage in various conditions.

SDC-1 is involved in various cellular processes, including cell proliferation, apoptosis, and inflammation. It plays a crucial role in modulating the vascular endothelial barrier function and inflammatory response, both of which are central to the pathophysiology of KD. SDC-1 interacts with several signaling molecules, including growth factors, cytokines, and chemokines, facilitating their binding to their respective receptors, thereby modulating downstream signaling pathways such as the MAPK/ERK^29^ and NF-κB pathways ^30^. These pathways are known to influence inflammatory processes and endothelial cell function, which are critical in the development of CALs.

In KD, the endothelial damage and increased vascular permeability contribute to the formation of coronary aneurysms. SDC-1 shedding, induced by pro-inflammatory cytokines, results in the release of its ectodomain, which can act as a soluble receptor, further exacerbating the inflammatory response and endothelial dysfunction. The loss of SDC-1 from the endothelial surface also leads to a breakdown of the glycocalyx, a protective layer on the endothelial cells, making the coronary arteries more susceptible to damage and aneurysm formation.

In our study, we have observed that elevated levels of SDC-1 are correlated with the severity of CALs in KD patients, suggesting that SDC-1 may serve as a potential biomarker for predicting the development and progression of CALs. The detailed mechanistic insights into SDC-1's role in KD provided in the revised manuscript aim to offer a more comprehensive understanding of its involvement in disease pathogenesis and its potential as a therapeutic target. (page 10-11, line 285-310)

**References**

29. Sun H, Hu Y, Gu Z, Owens RT, Chen YQ, Edwards IJ. Omega-3 fatty acids induce apoptosis in human breast cancer cells and mouse mammary tissue through syndecan-1 inhibition of the MEK-Erk pathway. Carcinogenesis. 2011 Oct;32(10):1518-24.

30. Lin L, Qiu D, Yang F, Xia Y, Cai S, Liao X, Deng W, Wu C. Hydrogen-rich saline upregulates the Sirt1/NF-κB signaling pathway and reduces vascular endothelial glycocalyx shedding in sepsis-induced acute kidney injury. Shock. 2024 May 27. (page 17, line 490-497)

*  Lack of consideration for confounding factors: The discussion does not address potential confounding factors, such as age, gender, disease severity, or medication use, that could influence the observed lipid and coagulation profiles in KD patients.

Reply: Thank you for your thorough review of our study, particularly the consideration of potential confounding factors. We fully concur on the significance of confounders in medical research and are grateful for your observation that our discussion section should further elaborate on this point.

In our study, we conducted statistical analyses on factors such as age, gender and weight, and found no statistically significant differences among the groups in our study. This suggests that the impact of these variables on the study outcomes within our sample may be limited.

We have expanded the discussion section to include descriptions of these statistical results and emphasized the importance of further exploring these factors in different research contexts. We believe that this transparent communication will aid readers in understanding our study findings and provide guidance for future research. Nonetheless, we recognize that disease duration, treatment status and medication use are also significant confounding factors in different samples or study designs. Therefore, we agree and suggest that the potential impact of these variables should continue to be monitored in future studies to ensure the accuracy and generalizability of the results. We have highlighted this in the limitations section.

Thank you again for your valuable comments, and we look forward to your further guidance.

**Changs in the text:**

In our study, we conducted statistical analyses on factors such as age, gender and weight, and found no statistically significant differences among the groups in our study. This suggests that the impact of these variables on the study outcomes within our sample may be limited. (page 11-12, line 330-333)

On the other hand, the diversity and variability of treatment status present challenges in controlling for this variable in the current analysis. What’s more, due to constraints on funding and resources, our study did not collect detailed data on disease duration. In future research, we will consider designing prospective studies to gather more comprehensive data, including regular updates on disease duration. We also plan to include treatment response as a covariate in the analysis to assess its potential impact on the outcomes. (page 12, line 340-346)

Figures
1.  Should add figures of Echocardiography or CT to illustrate how coronary arteries were assessed

Reply: Thank you for the valuable suggestion, which is highly appreciated. We have added the relevant figures of echocardiography as suggested.

**Changs in the text:**

CALs in this study were evaluated using echocardiography. The imaging was performed with a [GE Vivid E95, manufactured by GE Healthcare], which was utilized to obtain measurements of coronary artery dimensions. (Figure 2) (page , line )

Figure 2. The figures of echocardiography. A. Normal; B. Dilatation of the left main coronary artery; C. Dilatation of the left main coronary artery and the left anterior descending branch with thrombus formation; D. Dilatation of the right coronary artery; E. Localized aneurysm-like dilatation of the left anterior descending coronary artery. (page 18, line 507-510)

We have highlighted all changes made to the manuscript using tracked changes for your convenience.

Thank you once again for your constructive feedback, which has been invaluable in improving our manuscript. We hope that our revisions meet the expectations of the reviewers and the editorial board.

We look forward to your favorable response.

Best wishes,

Xiaoke Shang
